# Oncogene-mediated metabolic gene signature predicts breast cancer outcome

**DOI:** 10.1038/s41523-021-00341-6

**Published:** 2021-10-28

**Authors:** Merve Aslan, En-Chi Hsu, Fernando J. Garcia-Marques, Abel Bermudez, Shiqin Liu, Michelle Shen, Meredith West, Chiyuan Amy Zhang, Meghan A. Rice, James D. Brooks, Robert West, Sharon J. Pitteri, Balázs Győrffy, Tanya Stoyanova

**Affiliations:** 1 Department of Radiology, Canary Center at Stanford for Cancer Early Detection, Stanford University, Stanford, CA USA; 2 Department of Urology, Stanford University, Stanford, CA USA; 3 Department of Pathology, Stanford University, Stanford, CA USA; 4 TTK Lendület Cancer Biomarker Research Group, Research Centre for Natural Sciences, Institute of Enzymology, Magyar Tudósok Körútja, 1094 Budapest, Hungary; 5 Semmelweis University, Department of Bioinformatics and 2nd Department of Pediatrics, Tüzoltó Utca 7–9, 1094 Budapest, Hungary

**Keywords:** Breast cancer, Prognostic markers

## Abstract

Breast cancer remains the second most lethal cancer among women in the United States and triple-negative breast cancer is the most aggressive subtype with limited treatment options. Trop2, a cell membrane glycoprotein, is overexpressed in almost all epithelial cancers. In this study, we demonstrate that Trop2 is overexpressed in triple-negative breast cancer (TNBC), and downregulation of Trop2 delays TNBC cell and tumor growth supporting the oncogenic role of Trop2 in breast cancer. Through proteomic profiling, we discovered a metabolic signature comprised of TALDO1, GPI, LDHA, SHMT2, and ADK proteins that were downregulated in Trop2-depleted breast cancer tumors. The identified oncogene-mediated metabolic gene signature is significantly upregulated in TNBC patients across multiple RNA-expression clinical datasets. Our study further reveals that the metabolic gene signature reliably predicts poor survival of breast cancer patients with early stages of the disease. Taken together, our study identified a new five-gene metabolic signature as an accurate predictor of breast cancer outcome.

## Introduction

Breast cancer is the most common noncutaneous cancer among women and the second leading cause of cancer-associated deaths in women in the United States^1^. Triple-negative breast cancer (TNBC) is the most aggressive subtype of breast cancer, characterized by lack of estrogen receptor (ER), progesterone receptor (PR), or human epidermal growth receptor type 2 (HER2) expression. TNBC is associated with a higher rate of distant metastases, resistance to current therapies, and poor survival^2–4^.

To adapt to a nutrient-poor tumor microenvironment, by alterations in oncogenes and tumor suppressors cancer cells have the capacity to reprogram their metabolism via modulating key metabolic enzymes^5^. Metabolic reprogramming has been identified as a hallmark of cancer^6,7^. It is well established that highly proliferative cancer cells prefer glycolysis to produce energy even when the oxygen level is adequate for oxidative phosphorylation, also known as the “Warburg effect”^8–10^. Metabolic rewiring of cancer cells enables them to derive adequate energy and macromolecules for anabolic reactions even in nutrient-poor environments. TNBC is characterized by intensive glucose consumption, lower oxygen uptake, and elevated glycolysis compared to other breast cancer types as a result of highly elevated glycolytic enzymes and transporters^11–13^. TNBC also expresses a glycolytic gene signature with upregulated c-myc, a transcription factor orchestrating gene expression changes in molecular reprogramming^14,15^. Chemotherapy resistance may also be acquired through metabolic reprogramming of cancer cells^16^.

Tumor-associated calcium signal transducer 2 (TACSTD2), also known as a human trophoblastic cell surface antigen 2 (Trop-2, Trop2), is a transmembrane glycoprotein and an emerging candidate for targeted cancer therapies due to its overexpression in various epithelial cancers and its association with tumor metastasis and poor prognosis across multiple epithelial cancers^17–22^. Trop2 is overexpressed in breast cancer and in more than 85% of TNBC tumors^2,23^. High Trop2 levels are associated with poor survival in invasive ductal breast cancer patients, and membrane-localized Trop2 is an unfavorable prognostic marker for breast cancer patients^24–26^. Anti-Trop2 antibody–drug conjugate, Trodelvy (Sacituzumab govitecan-hziy) was recently approved by the FDA for treatment of metastatic refractory TNBC patients who have received at least two prior therapies^2^.

Here, we demonstrate that protein levels of Trop2 are highly elevated in breast cancer patients and downregulation of Trop2 by gene deletion or gene silencing significantly impairs TNBC cell growth and colony formation in vitro, and tumor growth in vivo. Likewise, Trop2 overexpression induces breast cancer cell growth, further highlighting the oncogenic role of Trop2 in the growth of TNBC. To delineate cellular changes mediated by Trop2, we evaluated global protein changes upon modulation of Trop2. Proteomic profiling of TNBC tumors with decreased levels of Trop2 via knockdown strategies revealed that several known oncogenic proteins and a metabolic cluster of proteins composed of TALDO1, GPI, LDHA, SHMT2, and ADK are significantly decreased. Consistent with this result, the expression levels of the identified oncogenes and five metabolic genes (TALDO1, GPI, LDHA, SHMT2, and ADK) are elevated in TNBC patients when compared to ER+ patient samples across multiple clinical datasets. More importantly, the identified five-gene (5-gene) metabolic signature predicts poor survival in patients with early-stage breast cancer. The 5-gene metabolic signature (TALDO1, GPI, LDHA, SHMT2, and ADK) correlates with poor overall and disease-free survival in 12 different mRNA expression clinical datasets. Collectively, these findings demonstrate that the oncogene-mediated 5-gene metabolic signature is a powerful marker for aggressive breast cancer and a predictor for inferior breast cancer survival.

## Results

### Trop2 is elevated in TNBC patients

**Fig. 1:**
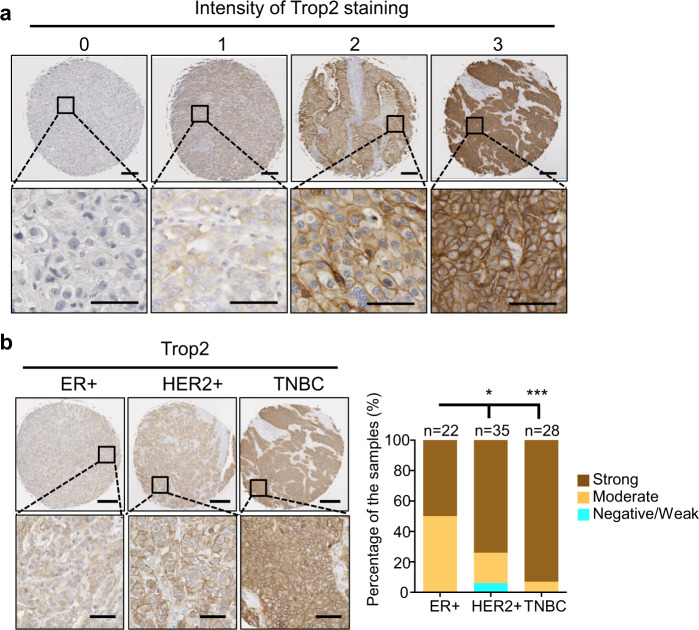
Trop2 is highly expressed in breast cancer. **a** Representative images of Trop2 staining intensity scores. Trop2 staining intensity is scored from 0 to 3. Scale bar represents 250 µm (upper panel) and 50 µm (lower panel). **b** Trop2 IHC staining on TMA including ER^+^ (*n* = 22), HER2^+^ (*n* = 35), and TNBC (*n* = 28) tissue samples. Representative images of ER+, HER2+, and TNBC samples (left) and Trop2 staining intensity distribution in ER+, HER2+, TNBC samples as percentages are demonstrated (right). Scale bar represents 250 and 50 µm for upper and lower panels. The statistical significance of the differences between population proportions was calculated by the normal distribution N (0,1) of the *Z*-score. Error bars represent standard deviation (SD). *P < 0.05, ***P < 0.001.

To evaluate Trop2 protein levels in independent patient cohorts, we analyzed Trop2 protein levels by immunohistochemistry (IHC) in ER+, HER2+, and TNBC samples using a tissue microarray (TMA) (Fig. 1a, b). The TMA contained 22 ER+ HER2−, 35 HER2+ (27 HER2+ ER−, and 8 HER2+ ER+), and 28 TNBC samples (Fig. 1a, b). High levels of Trop2, assessed by Trop2 intensity of IHC staining, occurred in 50% of ER+, 74% of HER2+, and 93% of TNBC samples (Fig. 1b). These results demonstrate that a high percentage of patients with TNBC have elevated levels of Trop2.

### Trop2 regulates TNBC tumor growth in vitro and in vivo

**Fig. 2:**
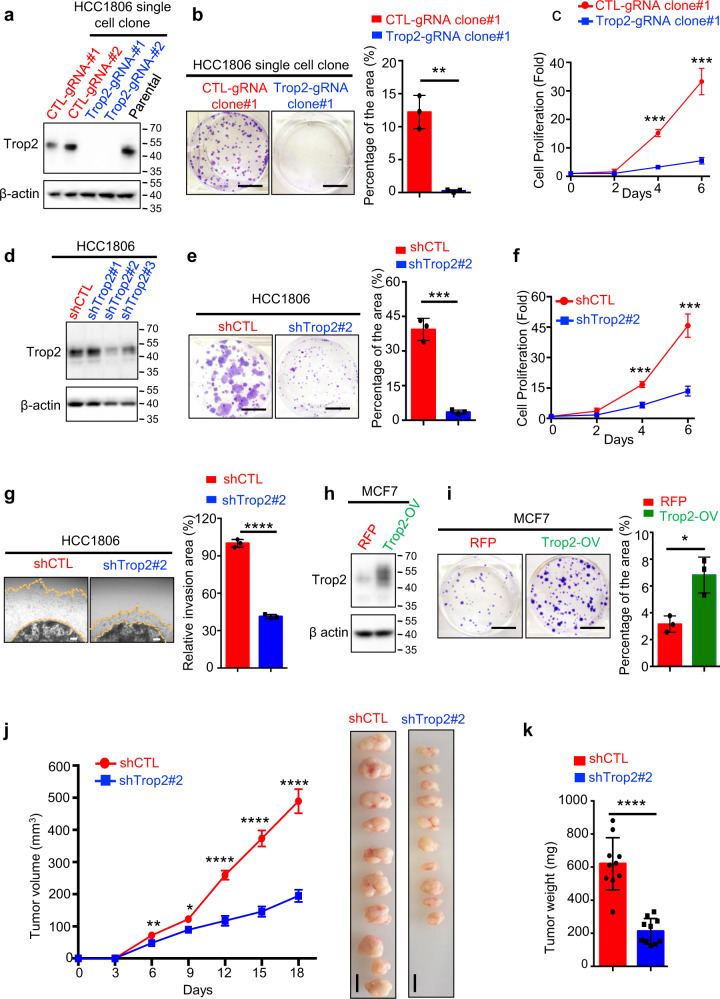
Trop2 regulates TNBC cell and tumor growth in vitro and in vivo. **a** Trop2 levels in HCC1806 control (CTL-gRNA-#1 and CTL-gRNA-#2), Trop2 gene deletion (Trop2-gRNA-#1 and Trop2-gRNA-#2), and parental cell lines were evaluated by western blot. The two blots were derived from the same experiment and were processed in parallel. The whole blots are shown in Supplementary Fig. 11a. **b** Colony formation assay of HCC1806 control and Trop2-gRNA-#1 cell lines. Representative images of wells after harvesting and staining with crystal violet (left) and quantification of percent area (right) are shown. The scale bar is 1 cm. Error bars represent SD. **c** Proliferation assay of HCC1806 control and Trop2-gRNA-#1 cells are presented as fold change over Day 0. Quantification measures cell count. **d** Trop2 levels in HCC1806 shCTL, and Trop2 knockdown cells (shTrop2#1, shTrop2#2, and shTrop2#3) were measured by western blot. The two blots were derived from the same experiment and were processed in parallel. The whole blots are included in Supplementary Fig. 11b. **e** Colony formation assays of HCC1806 shCTL and shTrop2#2 cells. Representative images of harvested wells stained with crystal violet (left) and the quantification of percent area (right) are shown. Scale bars represent 1 cm. Error bars represent SD. **f** Proliferation assay of HCC1806 shCTL and shTrop2#2 and cells, demonstrated as fold change based on the cell number of individual cell lines at Day 0. Quantification measures cell count and it is shown as fold change over Day 0. Error bars represent SD. **g** 3-D Matrigel drop invasion assay of HCC1806 shCTL and shTrop2#2. Representative images of the cells invaded area outside the drop (left), and quantification of percent invasion area relative to the shCTL (right) are demonstrated. Scale bars represent 250 µm. Error bars represent SD. **h** Trop2 protein levels in MCF7 cells stably expressing RFP or Trop2 and RFP were measured by western blot. The two blots were derived from the same experiment and were processed in parallel. The whole blots are shown in Supplementary Fig. 11c. **i** Colony formation assay of MCF7-RFP and MCF7-Trop2-OV. Representative images of harvested wells stained with crystal violet (left) and quantification of percent area (right) are shown. Scale bars represent 1 cm. Error bars represent SD. **j** Tumor volumes of HCC1806 shCTL (*n* = 10) and shTrop2#2 (*n* = 10) subcutaneously implanted xenografts in female NSG mice (left). Volumes measured every three days with calipers and quantified (length × width × height)/2. Tumor images at the experimental endpoint are shown (right). Error bars represent standard error (SE). **k** End of study tumor weights was measured and plotted (right). Scale bars represent 1 cm. Error bars represent SE. *P < 0.05, **P < 0.01, ***P < 0.001, and ****P < 0.0001 are derived from two-tailed Student’s *t* test.

To test the functional role of Trop2 in TNBC, we introduced *TROP2* gene deletion via CRISPR/Cas9 in HCC1806 TNBC cells that endogenously express Trop2 (Trop2-gRNA-1, Trop2-gRNA-2) (Fig. 2a). Loss of Trop2 significantly suppressed TNBC cell growth assessed by colony formation and proliferation assays (Fig. 2b, c and Supplementary Fig. 1a). To further confirm the oncogenic role of Trop2 in TNBC, we generated Trop2 knockdown HCC1806 TNBC cells using small hairpin RNA (shRNA) targeting Trop2 (Fig. 2d). Downregulation of Trop2 significantly impaired colony-forming ability and proliferation of HCC1806 TNBC cells (Fig. 2e, f and Supplementary Fig. 1b). Furthermore, downregulation of Trop2 dramatically decreased the invasion ability of the highly aggressive HCC1806 TNBC cell line measured by three dimensional (3-D) Matrigel drop invasion assay (Fig. 2g).

The functional role of Trop2 in breast cancer is further reinforced by a gain of function studies. Trop2 was overexpressed via lentiviral transduction in an ER+ breast cancer cell line, MCF7, characterized by low levels of endogenous Trop2 (Fig. 2h). Trop2 overexpression significantly increased the colony formation ability of MCF7 cells providing further evidence of Trop2 acting as an oncogene in breast cancer (Fig. 2i).

The oncogenic role of Trop2 in breast cancer was further tested in vivo (Fig. 2j, k and Supplementary Fig. 1c, d). HCC1806 TNBC cells with downregulation of Trop2 or *TROP2* gene deletion were subcutaneously implanted into the lateral flanks of female NSG mice, and tumor volumes were measured every three days. Downregulation or loss of Trop2 in HCC1806 TNBC cells led to a significant delay in tumor growth and a decrease in tumor weight (Fig. 2j, k and Supplementary Fig. 1c, d). Collectively, these results demonstrate the oncogenic role of Trop2 in breast cancer.

### Proteomic profiling reveals an oncogene-mediated metabolic signature in TNBC

**Fig. 3:**
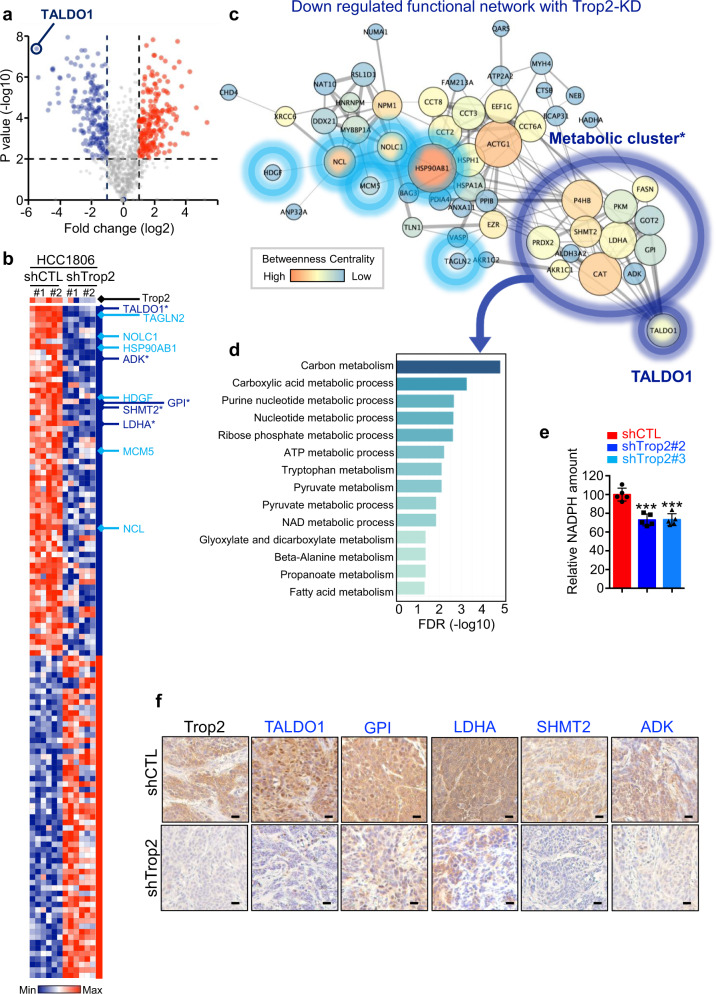
Trop2 knockdown modulates a set of metabolic proteins and known oncogenes in TNBC. HCC1806 shCTL and shTrop2 xenograft tumors (*n* = 2 per group) were analyzed in triplicate by liquid chromatography–tandem mass spectrometry (LC–MS/MS). **a** Volcano plot illustrating the increased and decreased proteins and dotted lines indicating *P* value < 0.01 and fold change > 2-fold. Blue represents decreased proteins; red represents increased proteins. **b** Heatmap demonstrating protein fold changes upon modulation of Trop2 (*n* = 64, fold change > 2, and *P* value < 0.01). Known oncogenic proteins (light blue), and metabolism-related proteins (dark blue, with stars) are indicated. In the heat map blue indicates decreased proteins; red indicates increased proteins. **c** Functional network analysis of decreased proteins upon Trop2 knockdown was performed by STRING (https://string-db.org/)^71^ and drawn with Cytoscape software 3.7.2.^28^ with yFiles Organic Layout. Proteins that belong to functional networks are shown. Top downregulated protein, TALDO1, is used to identify the associated metabolic cluster (* first neighbors of TALDO1). Known oncogenic proteins are noted with light blue circles, and the metabolic cluster is indicated in dark blue circles. The line thickness provides the strength of data support from the STRING database. The node size indicates neighborhood connectivity, and the node color represents betweenness centrality, which was generated from statistics of network analysis with Cytoscape software. **d** Functional enrichment analysis is summarized as a bar graph with false discovery rate (FDR) of Trop2 downregulated proteins in the Gene Ontology (GO). **e** NADPH quantification of HCC1806 shCTL, shTrop2#2, and shTrop2#3 cell lines. Relative luminescence represents the NADPH amount that is measured by NADP/NADPH-Glo^TM^ assay. Error bars represent SD, ***P < 0.001 derived from two-tailed Student’s *t* test. **f** IHC staining for Trop2, TALDO1, GPI, LDHA, SHMT2, and ADK in HCC1806 shCTL and shTrop2 xenografts. Scale bar represents 20 µm.

To delineate global protein changes mediated by Trop2 oncogene in TNBC, we performed liquid chromatography with tandem mass spectrometry (LC–MS/MS) proteomic profiling of tumor samples from HCC1806 shCtrl and shTrop2 xenografts (Fig. 3a, b, Supplementary Fig. 2 and Supplementary Table 1). Proteins that had more than five peptide counts and were downregulated more than twofold with *P* < 0.01 were included for further analysis. Functional protein association networks of 64 downregulated proteins were analyzed using STRING (https://string-db.org/) and were diagramed by Cytoscape software (Fig. 3c, d and Supplementary Fig. 3)^27,28^. Transaldolase 1 (TALDO1) and Transgelin 2 (TAGLN2) were the most significantly downregulated proteins in Trop2 knockdown HCC1806 tumors when compared to shCtrl (Fig. 3b). In addition, there were six significantly decreased oncogenes in Trop2 knockdown tumors including TAGLN2, NOLC1, HSP90AB1, HDGF, MCM5, and NCL (Fig. 3c).

Since TALDO1 is a metabolic protein and TAGLN2 is an oncogene, we further analyzed other metabolic and oncogenic proteins that were decreased upon Trop2 modulation (Fig. 3b, c). Interestingly, through functional network analysis, we identified a TALDO1-associated metabolic cluster of 13 proteins (TALDO1, GPI, SHMT2, LDHA, ADK, PRDX2, P4HB, PKM, FASN, GOT2, CAT, ALDH3A2, and AKR1C1) that were decreased in Trop2 knockdown tumors (Fig. 3c, d and Supplementary Fig. 3). The proteins in the TALDO1-associated metabolic cluster were enriched in various metabolic processes such as carbon metabolism, carboxylic acid, and nucleotide metabolic processes (Fig. 3c, d). Since nicotinamide adenine dinucleotide phosphate (NADPH) is synthesized by pentose phosphate pathway (PPP) and TALDO1 is a rate-limiting enzyme of PPP, we quantified NADPH levels in HCC1806 control and Trop2 knockdown cells as previously described^29^ (Fig. 3e). Consistent with the decrease in TALDO1 levels, we identified that NADPH has significantly decreased in Trop2 knockdown HCC1806 TNBC cells, suggesting the loss of Trop2 leads to a decrease in TALDO1 and NADPH levels (Fig. 3e). We further validated the expression levels of the top five proteins of the metabolic cluster, TALDO1, GPI, LDHA, SHMT2, and ADK, in HCC1806 xenografts. In line with the proteomics results, the top five decreased proteins in the metabolic cluster, TALDO1, GPI, LDHA, SHMT2, and ADK, were significantly decreased in HCC1806 xenografts upon Trop2 knockdown by IHC staining (Fig. 3f). These findings suggest that Trop2 may mediate metabolic reprogramming together with the induction of oncogenic proteins in TNBC.

### Oncogene-mediated metabolic gene signature is enriched in TNBC patients

**Fig. 4:**
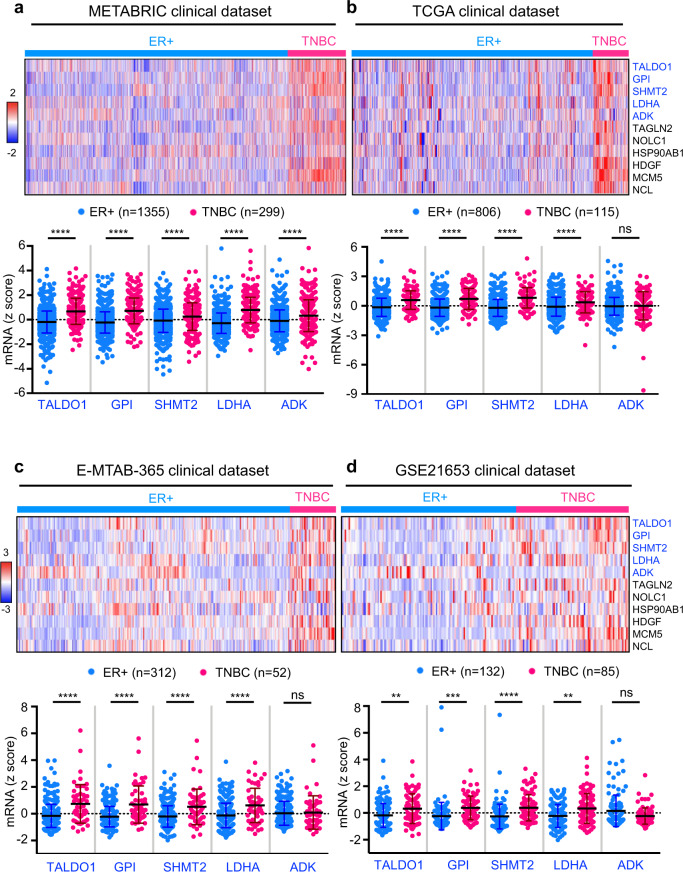
Five-gene metabolic signature is enriched in TNBC patients when compared to ER+ breast cancer patients. **a**–**d** Heatmap displaying mRNA levels of Trop2 associated 5-gene metabolic signature (TALDO1, GPI, SHMT2, LDHA, and ADK) and known oncogenes (TAGLN2, NOLC1, HSP90AB1, HDGF, MCM5, and NCL) in breast cancer patient samples (upper panel). The red color indicates a higher *z*-score whereas blue represents a lower *z*-score. Individual gene expression levels (TALDO1, GPI, SHMT2, LDHA, and ADK) are presented as dot plots (lower panel). The mRNA levels of the identified 5-gene metabolic signature in METABRIC ^57^ and TCGA (Firehose Legacy) clinical datasets are obtained from cBioPortal for Cancer Genomics (https://www.cbioportal.org/)^58,59^. The mRNA levels of the identified 5-gene metabolic signature in E-MTAB-365 ^60^ and GSE21653 ^61^ clinical datasets are shown. Error bars represent standard deviation. Adjusted *P* value was calculated based on Bonferroni testing in Prism software. **P < 0.01, ***P < 0.001, ****P < 0.0001, and ns not significant.

To analyze the clinical relevance of Trop2 oncogene-mediated proteome changes, 13 proteins that belonged to the TALDO1-associated metabolic cluster were further narrowed down to 7 metabolic proteins (TALDO1, GPI, SHMT2, LDHA, ADK, PRDX2, and CAT) based on the cut off of *p* value < 0.0005, fold change > 2 of the proteomic analysis (Supplementary Fig. 3). We evaluated transcript levels of *TALDO1*, *GPI*, *SHMT2*, *LDHA*, *ADK*, *PRDX2*, and *CAT* metabolic genes as well as the six oncogenes (*TAGLN2*, *NOLC1*, *HSP90AB1*, *HDGF*, *MCM5*, and *NCL*) in the METABRIC dataset (Fig. 4a and Supplementary Fig. 3). Transcript levels of five of the seven metabolic genes (*TALDO1*, *GPI*, *SHMT2*, *LDHA*, and *ADK*: 5-gene metabolic signature) and six oncogenes: *TAGLN2*, *NOLC1*, *HSP90AB1*, *HDGF*, *MCM5*, and *NCL*, were elevated in TNBC patients when compared to patients with ER+ breast cancer (Fig. 4 and Supplementary Figs. 3–5). *TALDO1*, *GPI*, *SHMT2*, and *LDHA* mRNA levels were higher in TNBC patient samples when compared to ER+ patient samples in three additional independent patient cohorts (Fig. 4 and Supplementary Fig. 5). *ADK* transcript levels were also significantly elevated in TNBC patients in the METABRIC cohort which has the largest sample size of the four datasets (Fig. 4). In the METABRIC cohort, the mRNA levels of the five individual metabolic genes and six oncogenes were higher in TNBC patients compared to either ER+ or HER2+ patients (Supplementary Fig. 4). Since the Trop2 protein levels do not correlate with the mRNA levels in patient datasets, Trop2 was not included in the mRNA analysis.

### Five-gene metabolic signature predicts poor outcome and worse overall survival in early-stage breast cancer

**Fig. 5:**
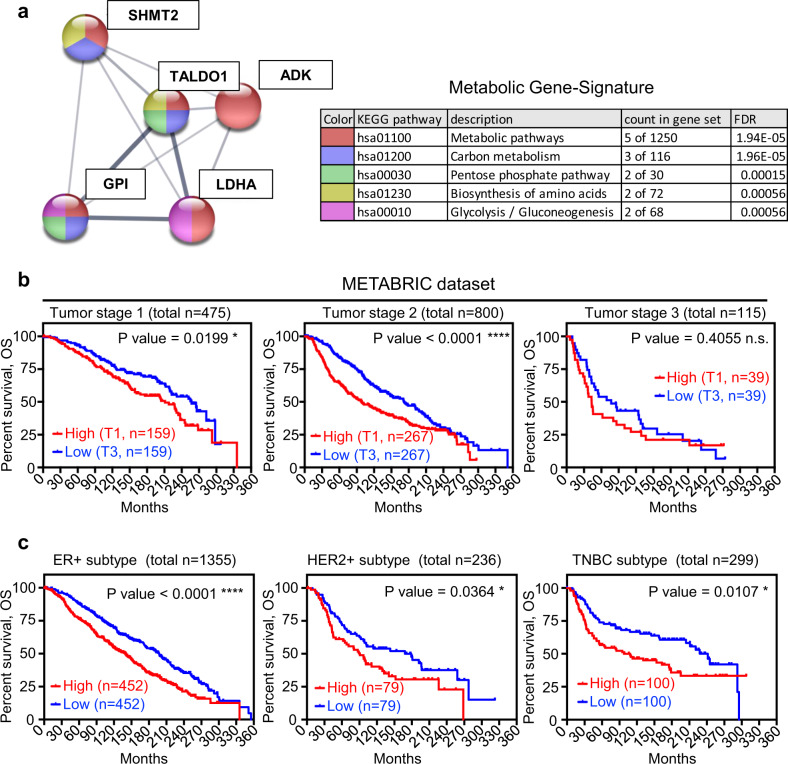
Five-gene metabolic signature predicts poor outcome in early stages of breast cancer independent of breast cancer subtypes. **a** Functional network analysis and functional enrichment analysis of five metabolic proteins. Functional network analysis is performed by String (https://string-db.org/)^71^. **b** Survival curves of breast cancer patients with tumor stage 1–3 samples that are categorized into two subgroups as upper tertile (T1) and lower tertile (T3) based on the mean mRNA *z*-score of 5 metabolic genes per sample from the METABRIC dataset. **c** Survival curves of patient subgrouping based on 5-gene metabolic signature with different subtypes of breast cancer including ER+, HER2+, and TNBC from METABRIC dataset. *P* value of survival analysis was calculated based on Log-Rank Test in Prism software. *P < 0.05, ****P < 0.0001 and ns not significant.

We further investigated the correlation of the 5-gene metabolic signature, summarized as a table from STRING analysis (Fig. 5a), with clinical outcomes to test whether this signature was associated with overall survival in early-stage breast cancer. mRNA expression levels of the 5-gene metabolic signature were analyzed in breast cancer patients with tumor stages I–III from the METABRIC dataset (Fig. 5b). We first categorized the patients based on the 5-gene metabolic signature expression levels and identified the lowest tertile (T3) in the low expression group versus high expression group encompassing the highest tertile (T1) and compared the overall survival of the identified groups in each tumor stage (Fig. 5B). Elevated levels of the 5-gene metabolic signature (T1) were associated with poor overall survival in stage I and stage II breast cancer (Fig. 5b), indicating the 5-gene metabolic signature may contain valuable prognostic information in early-stage breast cancer. Since the 5-gene signature is higher in TNBC, we analyzed outcomes in the defined molecular subtypes of breast cancer in METABRIC by sub-grouping patients into ER+, HER2+, and TNBC subtypes of breast cancer. For each of the molecular subtypes, the highest tertile of expression for the 5-gene metabolic signature (T1) was associated with poor overall survival (Fig. 5c). Furthermore, high expression of the 5-gene metabolic signature was associated with poor outcome in ER+ breast cancers in the E-MTAB-365 dataset, confirming that its prognostic value is not due to its association with TNBC (Supplementary Fig. 6).

### 5-gene metabolic signature predicts worse overall and disease-free survival in breast cancer patients

**Fig. 6:**
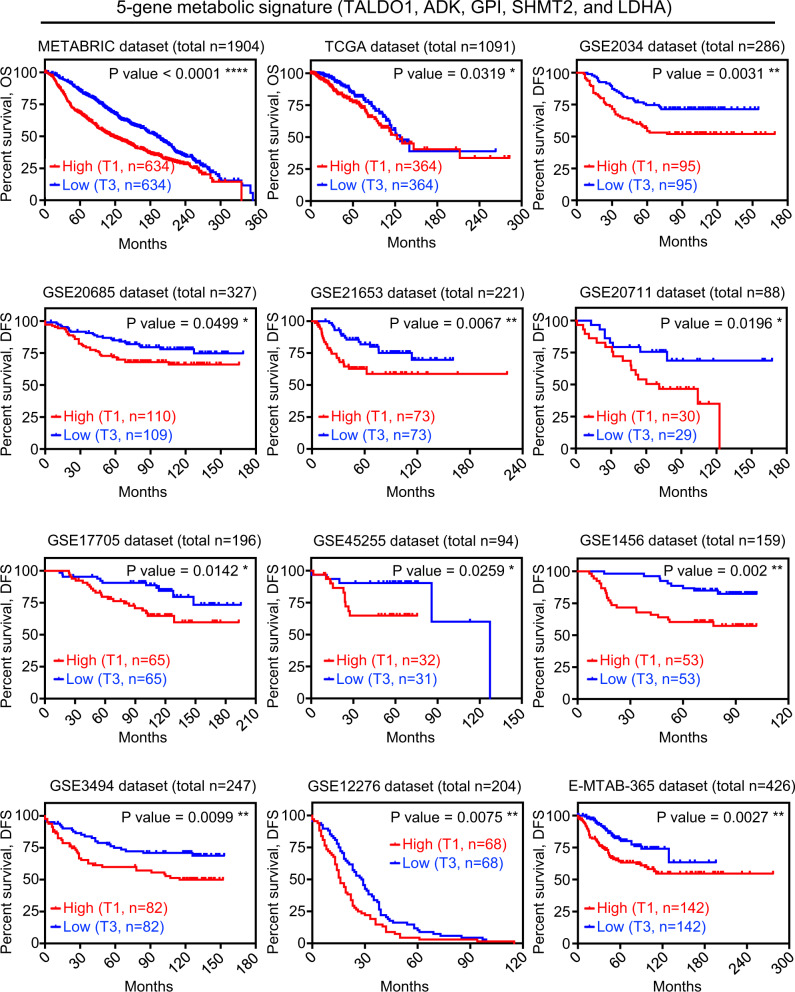
Five-gene metabolic signature predicts worse overall and disease-free survival in breast cancer patients. Survival curves (overall survival or recurrence-free survival) of breast cancer patients were sorted into two subgroups as upper tertile (T1) and lower tertile (T3) based on the mean of mRNA *z*-score of 5 metabolic genes per sample from 12 clinical public datasets. The overall survival (OS) information of METABRIC^57^ and TCGA (Firehose Legacy) clinical datasets are obtained from cBioPortal for Cancer Genomics^58,59^. The relapse-free survival (RFS) information of all other clinical datasets is generated using Kaplan–Meier Plotter (https://kmplot.com/analysis/index.php?p=service)^62^. *P* value of survival analysis was calculated based on Log-Rank Test in Prism software. *P < 0.05, **P < 0.01, ****P < 0.0001.

Next, we analyzed the correlation of the 5-gene metabolic signature with disease outcome in 12 independent mRNA expression breast cancer cohorts with long-term clinical follow-up were investigated by Kaplan-Meier analysis (Fig. 6). As seen in METABRIC, the tertile with high expression of the 5-gene metabolic signature (T1) in TCGA was associated with significantly worse overall survival when compared to the lowest (T3) (Fig. 6). High 5-gene metabolic signature (T1) also predicted worse disease-free survival in ten additional independent clinical cohorts of breast cancer patients (Fig. 6 and Supplementary Fig. 7 with complete T1–T3 plots). Furthermore, using median expression ranking of the 5-gene metabolic signature, patients who had higher expression levels of the 5-gene metabolic signature had shorter overall survival and shorter time to recurrence in nine different mRNA expression cohorts of breast cancer patients (Supplementary Fig. 8). In addition, elevated median and tertile expression levels of four out of the five genes in the 5-gene metabolic signature, *TALDO1*, *GPI*, *LDHA*, and *SHMT2* may provide benefit as individual prognostic predictors in breast cancer (Supplementary Fig. 9). Moreover, transcript levels for each gene alone compared to the five genes together showed that upregulation of the five metabolic genes together was a more powerful prognostic marker in 12 independent mRNA expression cohorts of breast cancer patients (Supplementary Fig. 10). Taken together, these findings describe a new 5-gene metabolic signature potentially mediated by oncogenic alterations in breast cancer. Most importantly, elevated intratumoral mRNA of 5-gene metabolic signature predicts worse disease outcomes at an early tumor stage of breast cancer.

## Discussion

Trop2 is elevated across epithelial cancers and commonly acts as an oncogene that promotes tumor growth and metastasis^18,19,30–42^. Consistent with these findings, here we demonstrate that Trop2 protein levels are elevated in TNBC when compared to ER+ and HER2+ patients. Furthermore, *TROP2* gene deletion and gene silencing suppressed TNBC cell growth in vitro and in vivo. Our findings suggest that Trop2 is a critical determinant of TNBC tumor growth. To gain insights into the global protein changes induced by Trop2, we further analyzed changes in protein levels upon modulation of Trop2. *TROP2* downregulation decreased the expression of TALDO1 associated metabolic clusters of proteins and a number of known oncogenic proteins. The analysis of four different clinical data sets revealed that expression levels of the identified 5 metabolic genes (*TALDO1*, *GPI*, *LDHA*, *SHMT2*, and *ADK*) and oncogenes (*TAGLN2*, *NOLC1*, *HSP90AB1*, *HDGF*, *MCM5*, and *NCL*) were consistently elevated in TNBC patients compared to the ER+ patients.

Among the genes in our discovered 5-gene metabolic signature are genes involved in breast cancer. TALDO1 is a rate-limiting enzyme of a non-oxidative branch of PPP and is found elevated in numerous cancers^43,44^. TALDO1 induces tumorigenesis amid oxidative stress by generating NADPH and ribose-5-phosphate that are essential for fatty acid and nucleic acid synthesis^45^. Glucose-6-phosphate isomerase (GPI) is the second glycolytic enzyme that drives the conversion of glucose-6-phosphate to fructose-6-phosphate^46^. It is also overexpressed in cancer by c-myc and HIF-1 induction resulting in glycolytic cancer phenotype^47^. Lactate dehydrogenase A (LDHA) is the enzyme in the final step of glycolysis that converts pyruvate to lactate in the cytoplasm^46^. LDHA is also elevated in TNBC, as are other glycolytic enzymes as a result of c-myc activity^48^. Interestingly, it is already shown that Trop2 overexpression induces c-myc expression through beta-catenin signaling^42^. Mitochondrial serine hydroxylmethyltransferase 2 (SHMT2), which has a role in the conversion of serine to glycine in the serine–glycine synthesis pathway, is significantly upregulated in breast cancer^49^. Moreover, increased SHMT2 correlates with poor disease outcomes mostly in ER-negative breast cancer^49^. Adenosine kinase (ADK) is also a key metabolic enzyme in the removal of adenosine that is tightly regulated in healthy cells. Adenosine signaling is associated with breast carcinoma and it is identified that downregulation of ADK decreases proliferation, migration, and invasion of TNBC cells, suggesting a functional role of ADK in TNBC^50^. Our study identified a new 5-gene metabolic signature (comprising of TALDO1, GPI, LDHA, SHMT2, and ADK) that is a more powerful prognostic marker than any of the single genes alone. Collectively, the identified 5-gene metabolic signature is related to oncogenic metabolism in breast cancer, and increased levels of the 5-gene metabolic signature correlate with tumor aggressiveness. Considering the well-established oncogenic role of Trop2 in various cancers including breast cancer, our studies suggest that Trop2 may potentially lead to an oncogene-mediated metabolic reprogramming in TNBC by regulating a group of metabolic genes and oncogenes.

Here, we demonstrate that elevated levels of 5-gene metabolic signature correlate with poor overall survival in early-stage breast cancer. Additionally, breast cancer patients with high expression levels of the 5-gene metabolic signature have worse overall and disease-free survival in 12 different mRNA expression clinical datasets of breast cancer patients. Furthermore, higher levels of the 5-gene metabolic signature collectively correlate with poor outcomes of breast cancer. Our study suggests that the 5-gene metabolic signature may represent a powerful tool for prognostic prediction in breast cancer. The defined 5-gene metabolic signature would be applicable for patients to assess the intratumoral expression levels of the identified 5-gene metabolic signature at the point of biopsy or surgery for outcome prediction. Most importantly, the 5-gene metabolic signature may serve as a valuable prognostic tool for breast cancer patients for the early stage of the disease.

## Methods

### Patient tissue samples

The TMA was purchased from Biomax (BR1505c) including 150 breast invasive ductal carcinoma cores from 75 different patients with duplicate cores per patient. The TMA had information regarding the clinical stages and IHC results of ER, PR, and HER2 hormone receptors. Patient samples were categorized based on their hormone receptor expression levels. Each case had two different cores that were blindly scored and averaged for Trop2 staining intensity. Ten out of 75 patient samples had different hormone receptor levels in each patient core. TMA has total *n* = 22 ER+ HER2−, *n* = 35 HER2+ (27 HER2+ ER−, and 8 HER2+ ER+), and *n* = 28 TNBC cases. The intensity of Trop2 staining was blindly scored from 0 up to 3 as shown in Fig. 1a. The average intensity of staining between the two cores was calculated and shown from 0 to 1.5 is negative/weak, equal and more than 1.5, and less than 2.5 is moderate, equal and more than 2.5 is strong. Trop2 staining intensity distribution of ER+, HER2+, and TNBC samples were plotted as percentages of the samples.

### Cell lines and culture

MCF7 was a kind gift from Dr. James Brooks’s laboratory at Stanford University (Palo Alto, CA). HCC1806 cells were purchased from ATCC. Cells were grown in RPMI supplemented with 10% fetal bovine serum, 1% penicillin/streptomycin, and 1% Glutamax. Cell culture was performed in a 37 °C incubators with 5% CO_2_. Warm Trypsin/EDTA (0.25%) was used for dissociation. All cells were authenticated through the Genetica cell line testing. Cells were tested for mycoplasma using Lonza Mycoalert Detection Kit (Lonza).

### Generation of control, Trop2 overexpression, or knockdown cell lines

FUCRW plasmid was a generous gift from Dr. Owen Witte’s laboratory at the University of California Los Angeles. The FUCRW-Trop2-OV construct generation was described previously^34^. Totally, 2 × 10^5^ MCF7 cells were infected with lentiviruses generated from the FUCRW or FUCRW-Trop2-OV constructs in a 6-well plate at a multiplicity of infection 5 with polybrene (10 μg/mL). Infection was confirmed by visualization of RFP positive cells under a fluorescence microscope 72 h post infection. Totally, 2 × 10^5^ HCC1806 cells were infected with viruses generated from pLKO.1-control scramble shRNA vector, or pLKO.1-Trop2 shRNA. Infected cells were selected for six days with puromycin (0.5 μg/mL) and Trop2 protein expression was confirmed with western blot. shRNA vector was a kind gift from David Sabatini (Addgene plasmid #1864; http://n2t.net/addgene: 1864; RRID: Addgene_1864). Trop2 pLKO.1 shRNA plasmids TRCN0000056419, target sequence GAGAAAGGAACCGAGCTTGTA (named shTrop2#1), TRCN0000056421, target sequence CGTGGACAACGATGGCCTCTA (named shTrop2#2), and TRCN0000303500, target sequence CGGCGCAAAGGAGACGTTTAT (named shTrop2#3) were purchased from Millipore Sigma (St. Louis, MO).

### Generation of control and Trop2 knockout cell lines

The generation of Trop2 knockout stable clone with the guide RNA sequence: CACCAGCGTGCGGGCGTTCT by CRISPR/Cas9 system was previously described^34^. Control double nickase plasmid from Santa Cruz (sc-437281) was used to generate stable control CTL-gRNA clones #1 and #2. Transient transfection of the knockout and control guide RNA constructs was performed using lipofectamine in HCC1806 cells. Cells were diluted to seed one cell per well in a 96-well plate after puromycin (0.5 μg/mL) selection for 6 days. Cells were expanded and cultured into two 96-well plates. Trop2 levels of each clone were detected through flow cytometry on the first plate by the Guava^®^ easyCyte Flow Cytometer (EMD Millipore). The selected successful Trop2 knock-out or control gRNA clones generated by CRISPR/Cas9 were expanded from the second plate for further experiments.

### Colony formation assay

Totally, 400 cells/well for MCF7 and 500 cells/well for HCC1806 cell lines were plated in 6-well plates in triplicates and cultured for 12 days with media changing every 3 days. The colonies were fixed with ice-cold methanol, stained with 0.01% crystal violet, and imaged. The images were analyzed with ImageJ and the percentage of the area was quantified and plotted.

### Cell proliferation assay

Totally, 1 × 10^4^ cells per well were plated in a 24-well plate in triplicates. The trypsinized cells were counted on days 2, 4, and 6 using trypan blue staining. The proliferation rate was plotted as a fold change based on the seeding cell number.

### Matrigel drop 3D invasion assay

Totally, 5 × 10^4^ cells were mixed with 10 μl Matrigel and plated in a 24-well plate as a single droplet in the middle of a well as it is described previously^34,51–53^. After 20 min of incubation at 37 °C, the Matrigel was solidified, and the media was added. The cells were grown for 6 days, and the media was changed every 3 days. The cells were imaged with a stereomicroscope (Leica), and cells that invaded the area outside of the drop were measured by ImageJ.

### Animal studies

All the procedures performed in this study were approved by Stanford Administrative Panel on Laboratory Animal Care (APLAC), IACUC. For all the studies, 6–8-week-old female NSG (NOD-SCID-IL2Rγ-null) mice (Jackson Laboratory) were used and housed at Stanford University animal facilities. The tumor volumes were calculated based on every third-day measurements of length (L), width (W), and height (H) with the formula: (L × W × H)/2. When the average tumor size reached 250 mm^2^ (Supplementary Fig. 1c) or 500 mm^2^ (in Fig. 2j), tumors were harvested and fixed at 10% formalin at 4 °C overnight. The tumors were processed and embedded in paraffin.

### Liquid chromatography and mass spectrometry analysis

Frozen xenograft tissues of two different tumors per group were homogenized in 800 µL of chilled lysis buffer consisting of 2% sodium dodecyl sulfate (SDS) (Thermo Fisher Scientific) and 1× protease inhibitor (Sigma Aldrich). Subsequently, samples were sonicated on ice with a Branson probe sonicator (Fisher Scientific) with the amplitude set at 40% for 15 s followed by 30 seconds off, with this cycle repeated three times. Then, lysate samples were centrifuged at 14,000*g* for 10 min at 4 °C. Extracted proteins were quantified by performing a standard bicinchoninic acid (BCA) protein assay (Thermo Fisher Scientific). Shotgun proteomics was performed on 25 µg of protein by reducing disulfide bonds with 5 µL of 200 mM tris (2-carboxyethyl) phosphine (TCEP) (Sigma Aldrich) in 100 µL 50 mM ammonium bicarbonate (Sigma Aldrich) and incubated at 65 °C for 1.5 h. The resulting free sulfhydryl groups on cysteines were alkylated by adding 7.5 µL of 200 mM iodoacetamide (Across Organics) with incubation at room temperature in the dark for 1 h. Then, proteins were precipitated by adding 1 mL of cold acetone and stored at −20 °C overnight. The following morning, precipitated proteins were pelleted by centrifugation under the same conditions described above. Pelleted proteins were digested with 1 µg of sequencing grade modified trypsin enzyme (Promega) in 100 µL of 50 mM ammonium bicarbonate buffer solution followed by incubation at 37 °C overnight without shaking. Tryptic peptides were dried using a speed vacuum system (LABCONCO) and re-constituted in 50 µL of 0.1% formic acid in water.

Reconstituted tryptic peptides were separated and analyzed by reversed-phase liquid chromatography on a Dionex UltiMate 3000 RSLC nano system coupled to an LTQ Orbitrap Elite mass spectrometer system (Thermo Fisher Scientific). Three technical replicates for each sample were performed. 1.5 µg of tryptic peptides were loaded onto a PepMap 100 C18 trap column (Thermo Fisher Scientific) at 5 µL/min for 10 min. Then, tryptic peptides were separated on a 25 cm analytical column (New Objective) packed with Magic C18 100 Å bedding material (Michrom Bioresources). The flow rate was held at 0.5 µL/min throughout the gradient. 10 min of loading time held buffer A (0.1% formic acid in water) and buffer B (0.1% formic acid in acetonitrile) at 98% and 2%, respectively. Buffer B was slowly increased to 35% over 100 min followed by a swift increase to 85% over 7 min with a 5 min hold time until returning to the initial condition of 2% in 2 min for column equilibration. The eluting peptides were ionized with 1.8 kV nano-ESI source voltage and the top 10 most abundant ions detected by the mass spectrometer were selected for collision-induced dissociation. The MS1 mass resolution was set to 60,000 with a mass scan range from 400 to 1800 m/z. The collision energy for MS/MS ion fragmentation was set to 35 eV, the AGC target set to 3e4, and dynamic exclusion enabled for 30 s.

### Proteomic data analysis

Each resulting LC–MS raw data were searched using Byonic 2.11.0 (Protein Metrics) twice against the corresponding taxonomy reference Swiss-Prot database. First, containing the human reference proteome (2017; 20,484 entries), and again using the mouse reference proteome (2017; 17,191 entries). Database searches were performed including trypsin digestion with a maximum of two missed cleavages, and mass tolerance of precursor and fragment ions were set to 0.5 Da and 10 ppm, respectively. Fixed cysteine carbamidomethylation, variable methionine oxidation, and asparagine deamination were also specified. Peptide identifications were filtered for a false discovery rate of 1%. Finally, peptides that overlapped in both human and mouse searches were removed to perform a conservative analysis of non-homologous only human identified proteins using an in-house R script, for each of the three technical replicates per experimental condition (HCC1806 shCTL, and HCC1806 shTrop2#2). Quantitative values were extracted from MS1 spectra from all resulting peptides using an in-house R script based on MSnbase package^54^ after chromatogram alignment. Using the AUC extracted, a pairwise relative quantification of each sample against the average of the corresponding controls was performed and analyzed using Generic Integration Algorithm, applying the principles of the WSPP model^55^ using SanXoT package^56^. Final statistical analysis was performed using Student’s *t* test, considering only proteins having more than 5 peptide counts, having a *P* value < 0.01, and a fold-change greater than 2 as significant.

### Immunoblotting

The cells were scraped from the plate with 1× phosphate-buffered saline (PBS) and lysed in RIPA lysis buffer supplemented with phosphatase and proteinase inhibitors. The protein concentration was measured with BCA assay, and the samples were denatured after 4× Laemmli buffer addition at 95 °C for 5 min. Samples were loaded and run on Novex Tris-Glycine gels (Invitrogen) and transferred onto a nitrocellulose membrane. The membrane was blocked in 5% milk in Tris-buffered saline (TBS) for 1 h at room temperature and was incubated in the primary antibodies in 1×TBST containing 0.1% tween-20 overnight at 4 °C. The membrane was washed and incubated with a secondary antibody (Thermo Fisher Scientific, PI31432) for 1 h at room temperature in 5% milk in TBS. After subsequent washing, the chemiluminescent signal was developed using Pierce ECL Western Blotting Substrate (Thermo Fisher Scientific) on an IVIS Lumina imager. Anti-Trop2 biotinylated antibody BAF650 (R&D), anti-transaldolase antibody (H-4) sc-166230, anti- LDH-A antibody (E-9) sc-137243, anti-mSHMT antibody (F-11) sc-390641, anti-ADK antibody (H-1) sc-514588, and anti-GPI Antibody (H-10) sc-365066 were used at 1:1000 dilution.

### Immunohistochemistry

Formalin-fixed paraffin-embedded tissue slides were deparaffinized by heating to 65 °C for 1 h and treatment with clarify and then hydrated. Sodium citrate buffer (10 mM/L, pH 6) was applied for all antibodies except LDHA for the antigen retrieval. 1 mM EDTA with 0.05% Tween 20 (pH 8.0) was used for LDHA detection. Antigen retrieval buffers were applied in a steamer at 95 °C for 20 min. Slides were then washed with water and treated with 3% hydrogen peroxide solution to inhibit endogenous peroxidase activity for 10 min. The tissues were blocked in 2.5% goat serum in PBS for mouse primary antibodies or 2.5% horse serum in PBS for rabbit primary antibodies. Primary antibodies were incubated overnight at 4 °C in a humidified chamber. Secondary horseradish peroxidase (HRP) antibodies (Vector Laboratories) were applied for 1 h after washing and then the tissues were stained with DAB (Dako Laboratories, Agilent). After the hematoxylin counterstaining, the tissues were dehydrated, air-dried, and coverslipped. The TMA images were taken with Hamamatsu nanozoomer and breast cancer patient tissue slides were imaged under 20× and 63× magnification with a Leica DMI microscope. Anti-Trop2 B9 sc-376746 (Santa Cruz) antibody was used in 1:100 dilution for TMAs IHC and anti-human Trop2 biotinylated antibody BAF650 (R&D) in 1:50 dilution was used for xenograft IHC staining. Antibodies against transaldolase (H-4) sc-166230, LDH-A (E-9) sc-137243, mSHMT (F-11) sc-390641, and ADK (H-1) sc-514588 from Santa Cruz Biotechnology, and GPI (#A6916) from ABclonal were used in 1:100 dilution.

### NADPH quantitation

Totally, 5 × 10^3^ cells were seeded per well in a 96-well plate overnight. Totally, 50 µl of NADP/NADPH-Glo^TM^ (Promega) detection reagent was added into each well that had the cells in 50 µl of media. The reductase in the detection reagent converted the proluciferin reductase substrate to luciferin in the presence of NADPH molecules. The plate was incubated for 60 min at room temperature and the luminescent signal, which was proportional to the NADPH amount, was measured by Tecan luminometer.

### Acquisition of gene expression and clinical data

The mRNA *z*-score data, overall survival information, ER/HER2/PR status, and tumor stages of METABRIC^57^ and TCGA Firehose Legacy clinical datasets, which were imported from the original TCGA Data Coordinating Center via the Broad Firehose (https://gdac.broadinstitute.org/, doi:10.7908/C11G0KM9) were obtained from cBioPortal for Cancer Genomics (https://www.cbioportal.org/)^58,59^. The mRNA raw data and ER/HER2/PR status in E-MTAB-365^60^ and GSE21653^61^ clinical datasets were used to generate the Kaplan–Meier Plotter (https://kmplot.com/analysis/index.php?p=service)^62^ and then normalized to *z*-score based on the equation: (raw score − population mean)/population standard deviation. Heatmaps of mRNA *z*-score in Fig. 3 were generated via Morpheus (https://software.broadinstitute.org/morpheus/). Relapse-free survival information of Kaplan–Meier plots of E-MTAB-365 (*n* = 426)^60^, GSE21653 (*n* = 230)^61^, GSE2034 (*n* = 286)^63^, GSE20685 (*n* = 327)^64^, GSE20711 (*n* = 88)^65^, GSE17705 (*n* = 196)^66^, GSE45255 (*n* = 94)^67^, GSE1456 (*n* = 159)^68^, GSE3494 (*n* = 249)^69^, GSE12276 (*n* = 204)^70^ were collected from Kaplan–Meier Plotter (https://kmplot.com/analysis/index.php?p=service)^62^ and then Log Rank Test of survival analysis was performed in Prism software. *N* numbers indicate the number of patients with the follow-up survival data available of the datasets.

#### Patient cohorts

METABRIC includes 1355 ER+ and 299 TNBC samples, TCGA contains 806 ER+, and 115 TNBC samples, E-MTAB-365 includes 312 ER+ and 52 TNBC samples and GSE21653 includes 132 ER+ and 85 TNBC samples. The mRNA *z*-scores for the indicated genes from the clinical datasets were downloaded and heatmaps were generated as a comparison of ER+ breast cancer and TNBC.

### Statistical analysis

The differences between population proportions were calculated by *z*-score test for two population proportions. In vitro and in vivo functional assays unless otherwise mentioned were analyzed by a two-tailed Student’s *t* test. An adjusted *P* value of gene expression levels between ER+ subtype versus TNBC subtype was calculated based on Bonferroni testing in Prism software. Survival analysis was calculated based on Log-Rank (Mantel–Cox) Test in Prism software.

### Reporting summary

Further information on research design is available in the Nature Research Reporting Summary linked to this article.

## Supplementary information

Supplementary Information

Reporting Summary

## Data Availability

The datasets generated during and/or analyzed during the current study are either included in the paper or available from the corresponding author on reasonable request. The proteomics data were deposited in PRoteomics IDEntifications (PRIDE), accession code PXD028335. Aggregate gene expression data analyzed in this study are available from the corresponding author on reasonable request. All requests should be directed to Dr. Tanya Stoyanova.
