# Supplementary material for: Oncogene-mediated metabolic gene signature predicts breast cancer outcome
Source: NPJ Breast Cancer. 2021 Oct 28;7:141. doi: 10.1038/s41523-021-00341-6 (PMC8553750; doi:10.1038/s41523-021-00341-6)
Supplement: Supplementary file 1 — Supplementary Information [file 41523_2021_341_MOESM1_ESM.pdf]

SUPPLEMENTARY INFORMATION

FOR

Oncogene-Mediated Metabolic Gene Signature Predicts Breast Cancer Outcome

Merve Aslan[1¶], En-Chi Hsu[1¶], Fernando J. Garcia Marques[1], Abel Bermudez[1], Shiqin Liu[1], Michelle Shen[1], Meredith West[2], Chiyuan Amy Zhang[2], Meghan A. Rice[1], James D. Brooks[2], Robert West[3], Sharon J. Pitteri[1], Balázs Győrffy[4, 5] and Tanya Stoyanova[1]

1. Department of Radiology, Canary Center at Stanford for Cancer Early Detection, Stanford University, Stanford, CA, USA. 2. Department of Urology, Stanford University, Stanford, CA, USA 3. Department of Pathology, Stanford University, Stanford, CA, USA. 4. TTK Lendület Cancer Biomarker Research Group, Research Centre for Natural Sciences, Institute of Enzymology, Magyar Tudósok körútja, 1094 Budapest, Hungary. 5. Semmelweis University, Department of Bioinformatics and 2nd Department of Pediatrics, Tüzoltó utca 7-9, 1094 Budapest, Hungary.

Supplementary Figure 1

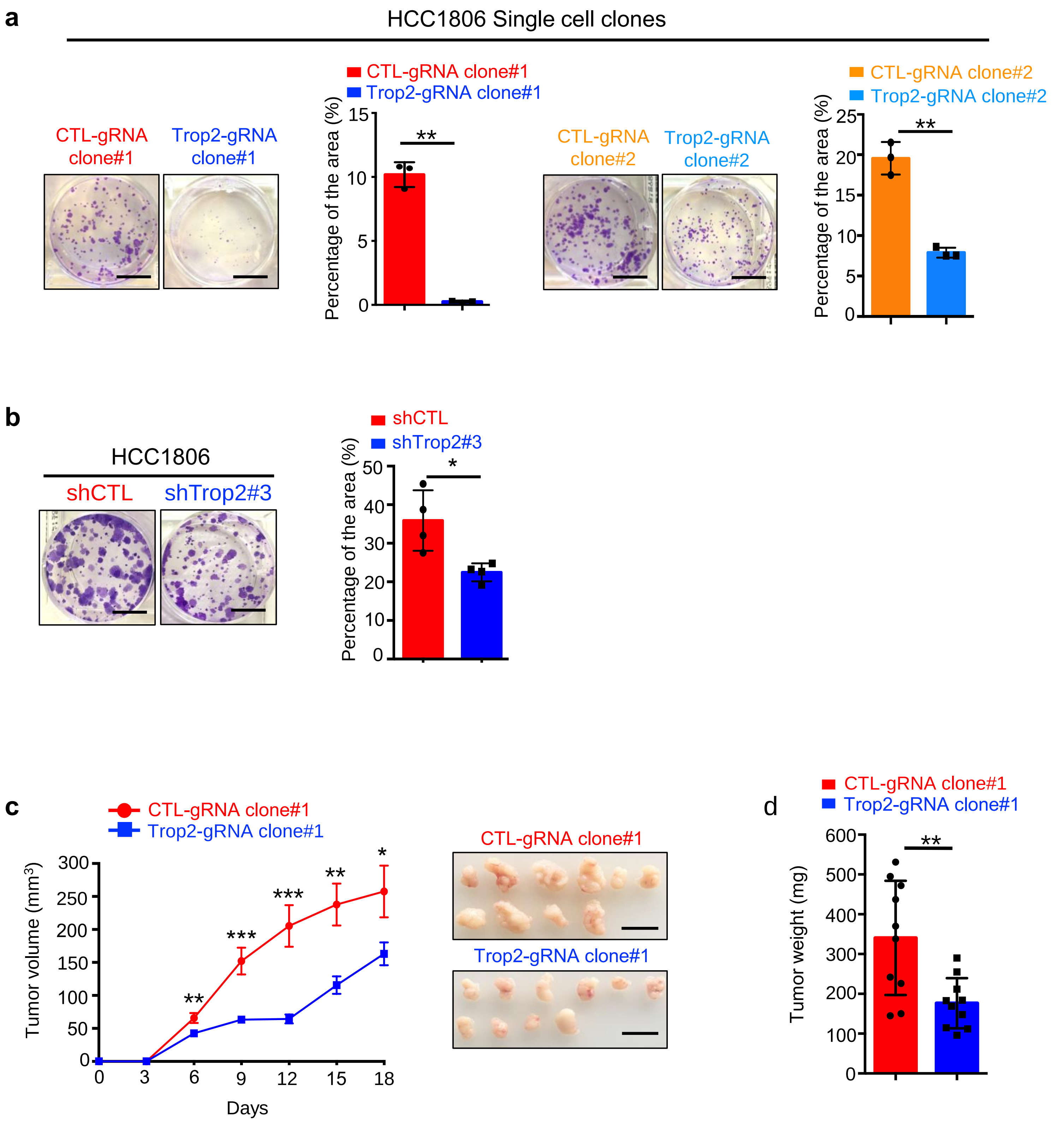

Supplementary Figure 1. Trop2 regulates TNBC growth *in vitro* and tumor growth *in vivo* (related to Figure 2). (a) Colony formation assay of HCC1806 CTL-gRNA-clone#1 and Trop2-gRNA-clone#1 (left) and HCC1806 CTL-gRNA-clone#2 and Trop2-gRNA-clone#2 cell lines (right). Representative images of wells after harvesting and staining with crystal violet, and quantification of percent area are shown. (b) Colony formation assay of HCC1806 shCTL and shTrop2#3 cell lines. Representative images (left) and quantification of percent area (right) are shown. Scale bars are 1 cm. Error bars represent SD. (c) Tumor growth curve and (d) tumor weights at the day of harvesting and tumor images of HCC1806 CTL-gRNA-clone#1 (n=10) and Trop2-gRNA-clone#1 (n=10) xenografts. Tumor volumes were measured every three days and calculated (length x width x height)/2. Scale bars are 1 cm. Error bars represent SE. * $P < 0.05$, ** $P < 0.01$, and *** $P < 0.001$ using two tailed Student's *t* test.

Supplementary Figure 2

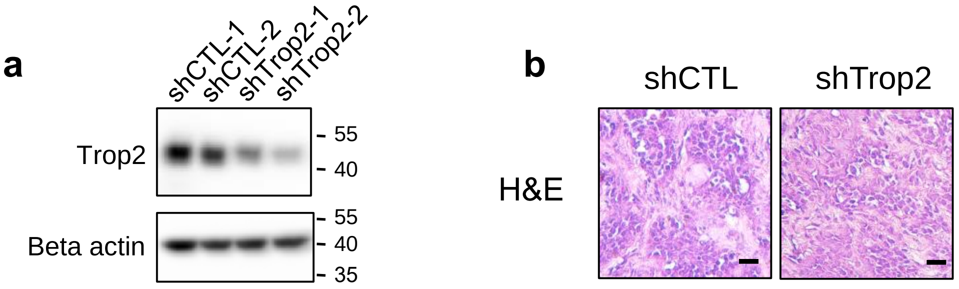

Supplementary Figure 2. Trop2 levels and histology of Trop2 knockdown xenografts (related to Figure 3). (a) Trop2 and beta actin expression levels detection in two different xenografts of HCC1806 shCTL and shTrop2#2 used for proteomic analyses by western blot. The two blots were derived from the same experiment and were processed in parallel. Whole blots are shown in Supplementary Figure 11d. (b) H&E staining of HCC1806 shCTL and shTrop2 xenografts.

Supplementary Figure 3

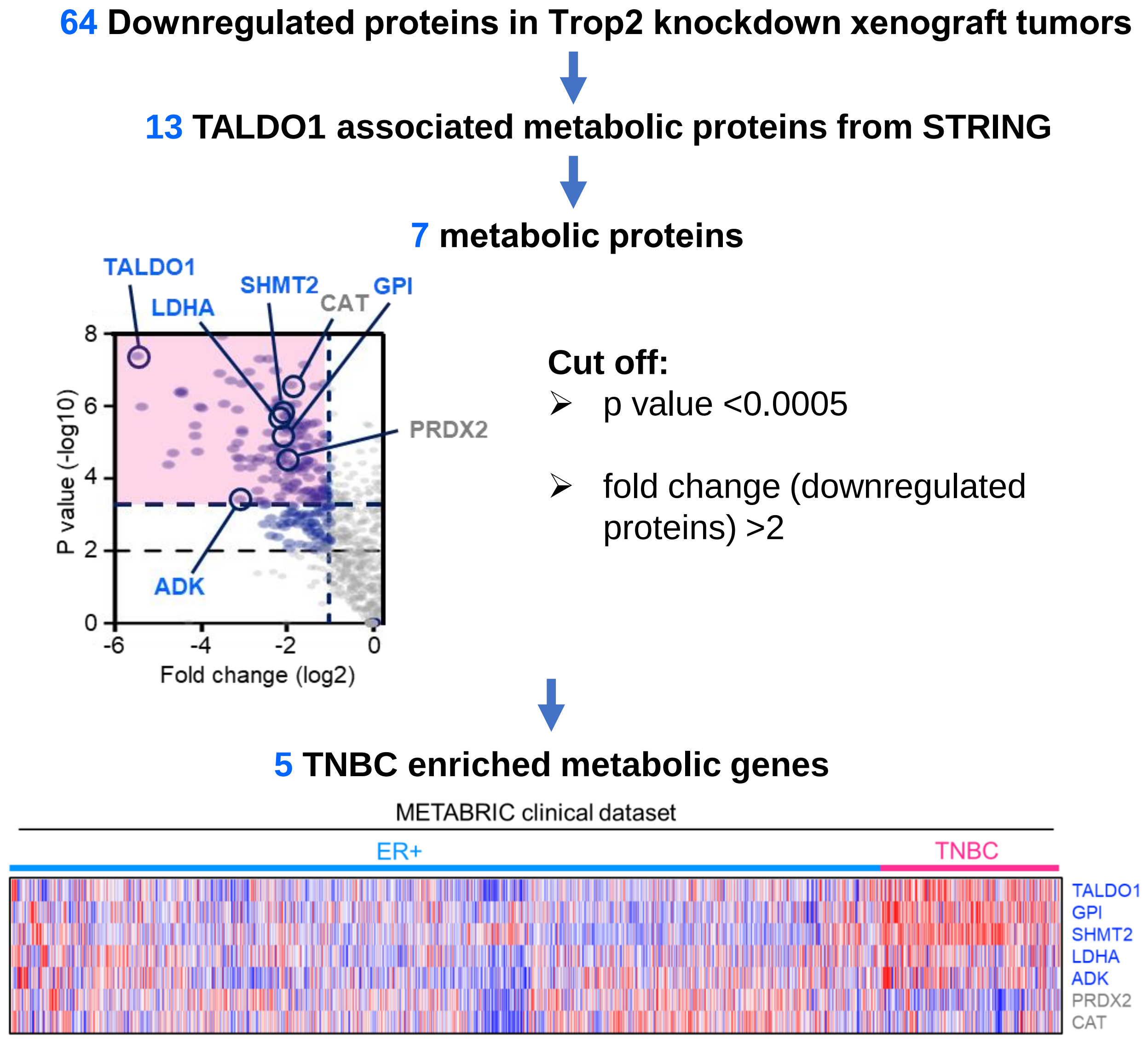

Supplementary Figure 3. Flow chart of candidate selection (related to Figure 3). 64 candidate proteins downregulated by Trop2 were identified utilizing *in vivo* tumor proteomic profiling. 13 metabolic proteins were further determined using STRING functional analysis database (Figure 3). 7 metabolic proteins (TALDO1, GPI, SHMT2, LDHA, ADK, PRDX2, and CAT) were selected based on more stringent cut off (fold change > 2 and p value < 0.0005). TALDO1, GPI, SHMT2, LDHA and ADK but not PRDX2 and CAT were enriched in TNBC in METABRIC clinical dataset. TALDO1, GPI, SHMT2, LDHA, ADK were selected as 5 metabolic genes for further analyses.

Supplementary Figure 4

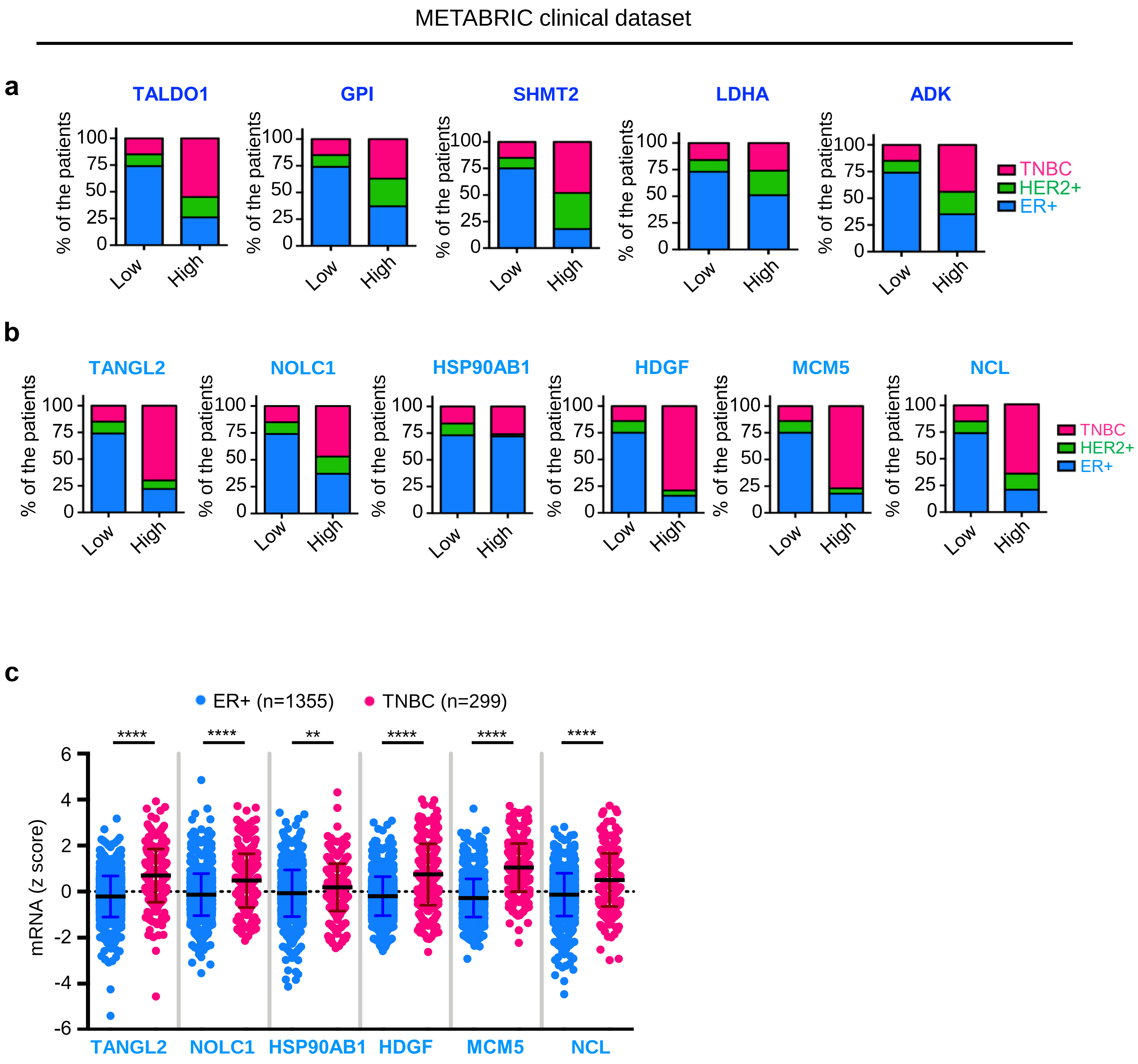

Supplementary Figure 4. Decreased metabolic proteins and known oncogenic proteins upon Trop2 knockdown are elevated in TNBC patient samples (related to Figure 4). (a) Trop2 related metabolic genes expression levels in ER+, HER2+ and TNBC patients from METABRIC clinical dataset (bar graphs). Patients were classified as mRNA z score high (more than 2) and low (less than 2) for each gene expression and the percentage of ER +, HER2+, or TNBC patients in each group is displayed. (b) Trop2 related known oncogenes expression levels in ER+, Her2+, and TNBC patients from METABRIC clinical cohort (bar graph). (c) Trop2 mediated known oncogenes expression levels are shown in ER+ and TNBC patients from METABRIC dataset (dot blot). Error bars represent standard deviation. Adjusted *P* value ,** $P < 0.01$ and **** $P < 0.0001$, was determined by Bonferroni testing.

Supplementary Figure 5

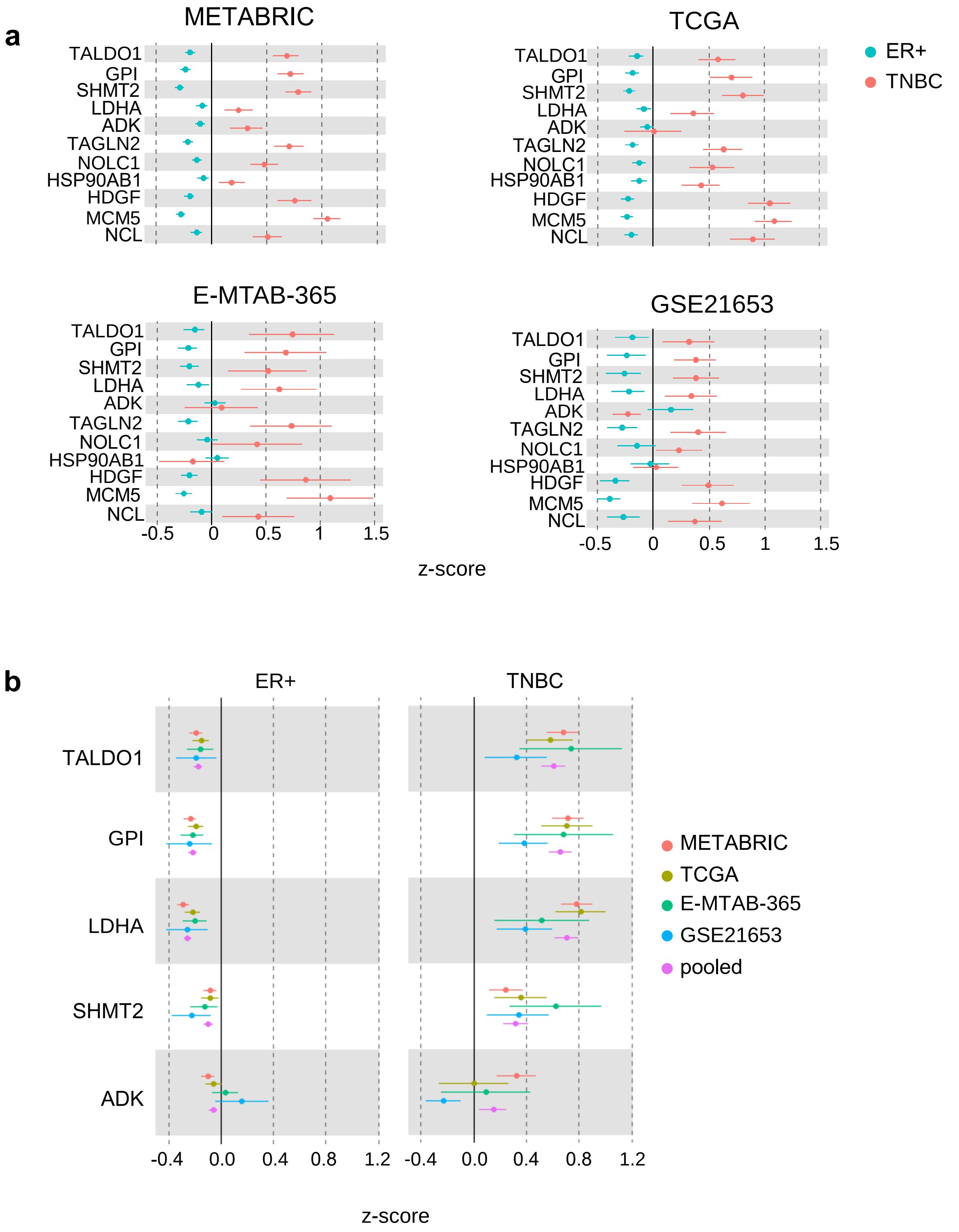

Supplementary Figure 5. Downregulated metabolic proteins and oncogenic proteins upon Trop2 knockdown are elevated in TNBC patient samples in four different datasets (related to Figure 4). (a) Five metabolic genes and six oncogenes mRNA z-score levels were analyzed in ER+ and TNBC patients in four different cohorts and presented by forest plot. (b) Five metabolic gene expression levels in four different patient datasets separately and four datasets pooled together were demonstrated by a forest plot. Graphs were plotted in forestplot in R, displaying the mean as a dot and the 95% confidence interval as a line through the mean.

Supplementary Figure 6

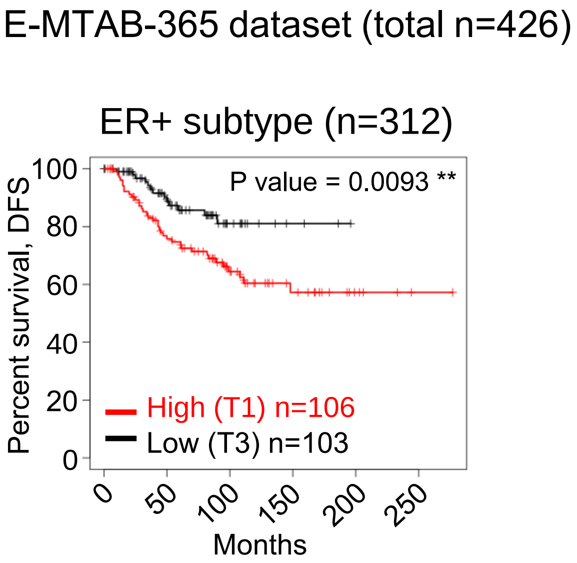

Supplementary Figure 6. 5-gene metabolic signature is prognostic in ER+ subtype in breast cancer (related to Figure 5C). Survival plots (relapse-free survival) of ER+ breast cancer patients from E-MTAB-365 clinical datasets that are sorted as tertiles into three subgroups (T1, T2 and T3) based on average mRNA value of 5 metabolic genes per sample from Kaplan-Meier Plotter (https://kmplot.com/analysis/index.php?p=service).

Supplementary Figure 7

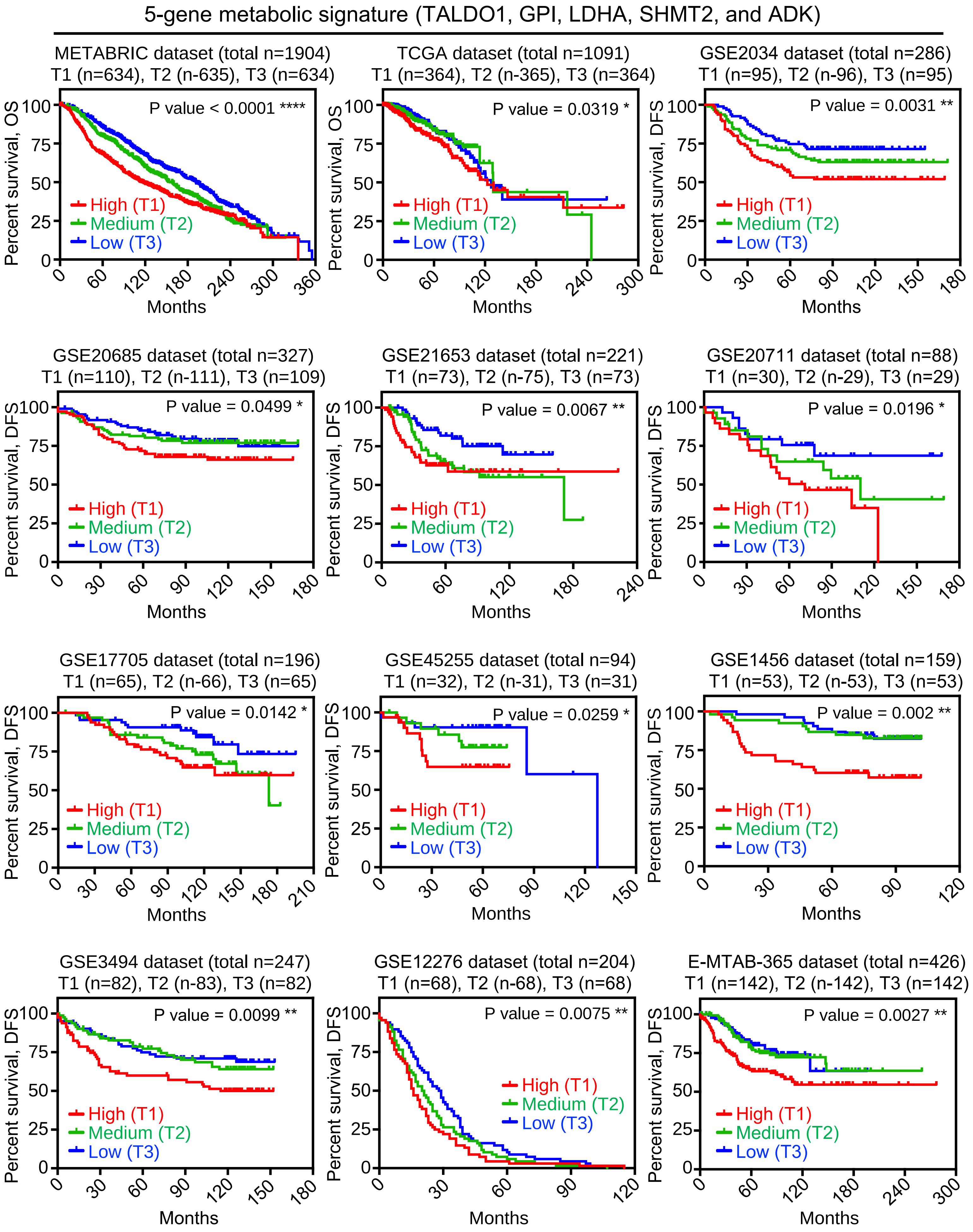

Supplementary Figure 7. 5-gene metabolic signature is prognostic in breast cancer (related to Figure 6). Survival plots (overall survival or relapse-free survival) of breast cancer patients from 12 clinical datasets shown in Figure 6 were sorted as tertiles into three subgroups (T1, T2 and T3) based on average mRNA value of 5 metabolic genes per sample.

Supplementary Figure 8

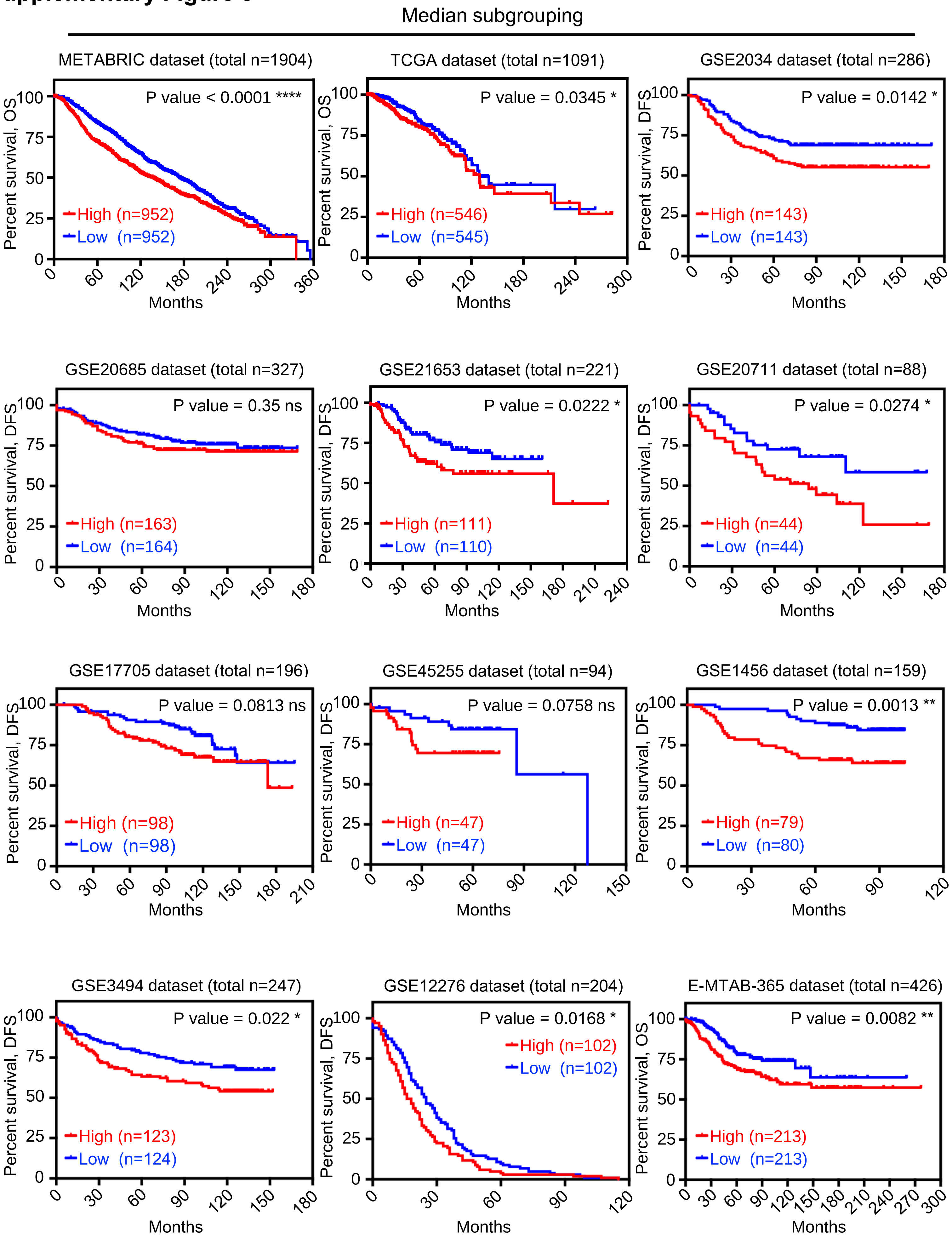

Supplementary Figure 8. 5-gene metabolic signature is prognostic in breast cancer (related to Figure 6). Survival plots (overall survival or relapse-free survival) of breast cancer patients from 12 clinical datasets that are sorted to two subgroups based on median of average mRNA value of 5 metabolic genes per sample. P values for the survival analysis were calculated based on Log-Rank Test. * $P < 0.05$, ** $P < 0.01$, **** $P < 0.0001$, and ns = not significant.

Supplementary Figure 9

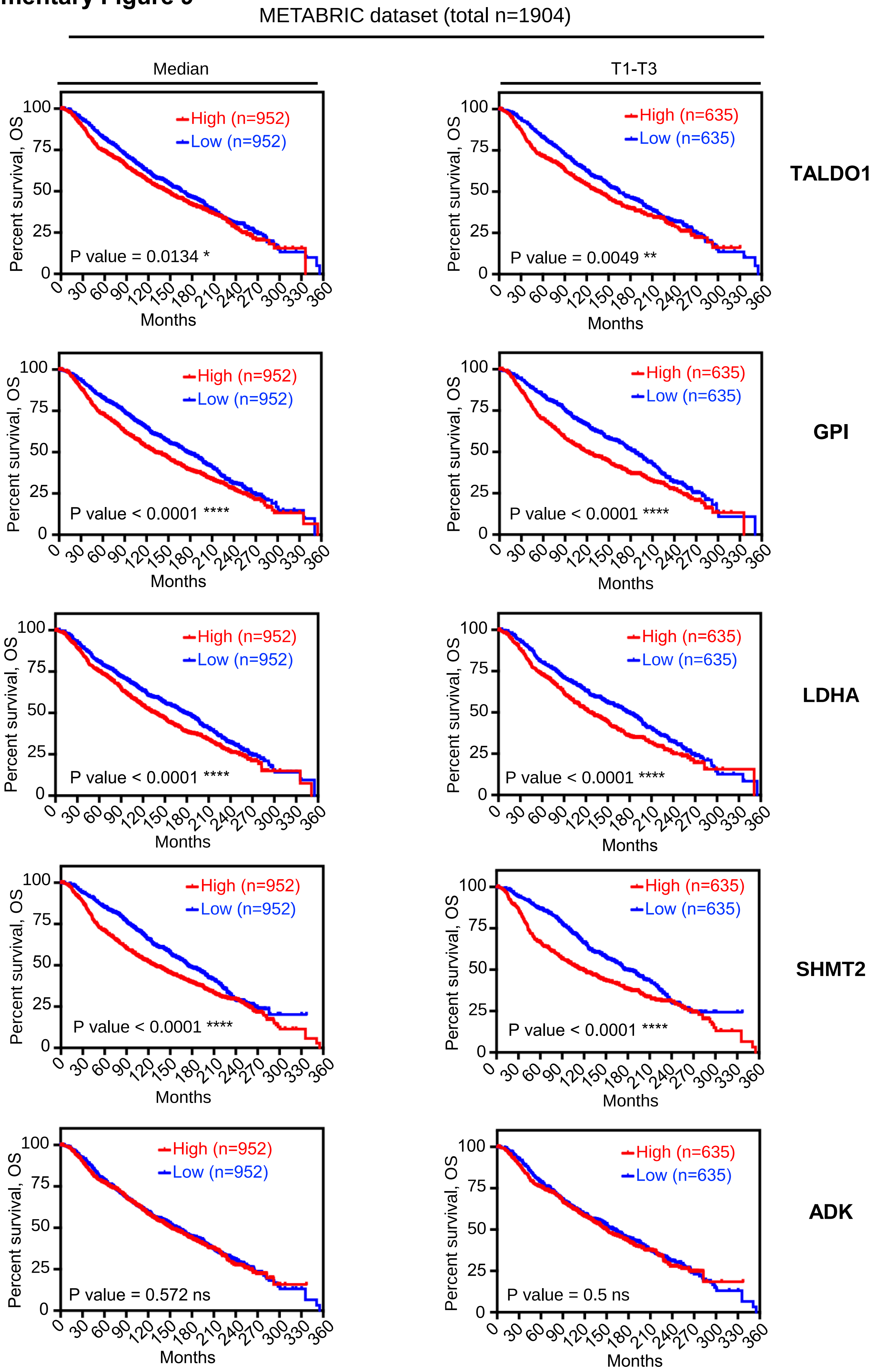

Supplementary Figure 9. Survival analysis of individual genes of the metabolic signature (Related to Figure 6). Survival curves of breast cancer patient samples that are subgrouped by median or tertile expression levels of each metabolic gene (TALDO1, GPI, LDHA, SHMT2, and ADK) in METABRIC clinical dataset. P value of survival analysis is calculated based on Log-Rank Test. * $P < 0.05$, ** $P < 0.01$, **** $P < 0.0001$, and ns = not significant.

Supplementary Figure 10

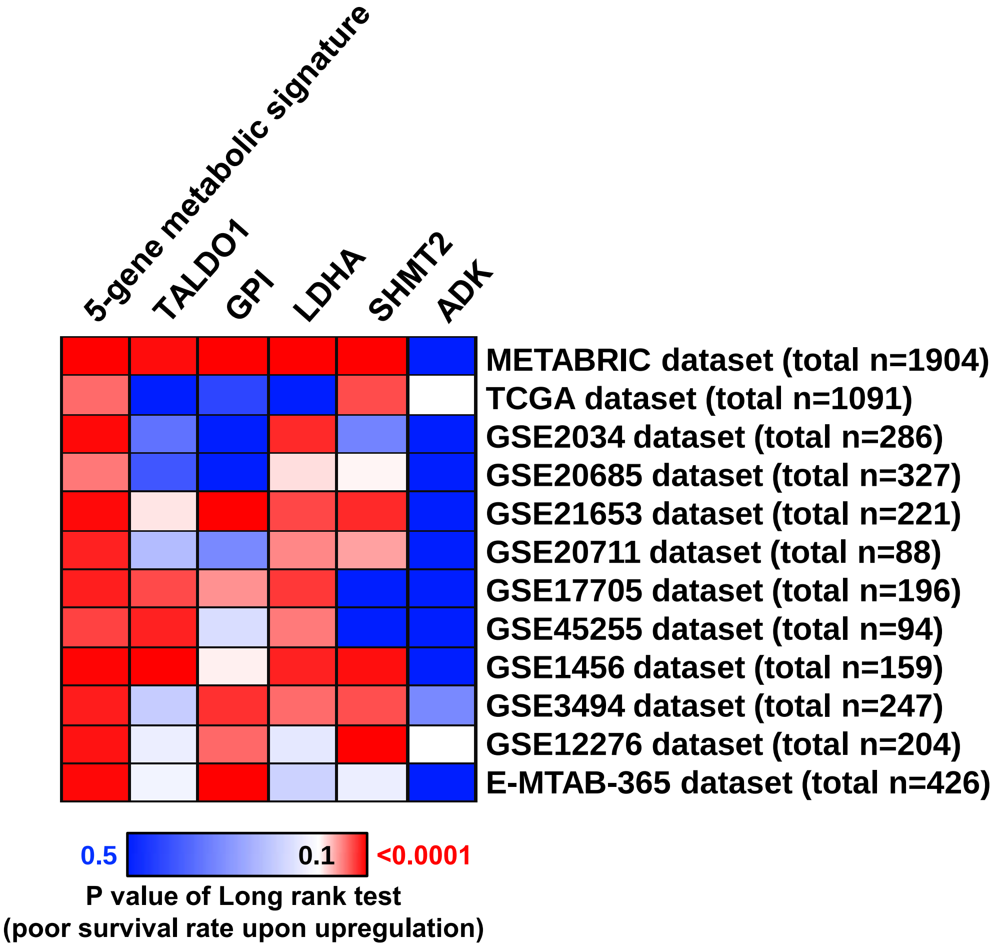

Supplementary Figure 10. 5-gene metabolic signature correlates with worse outcome in breast cancer when compared to each gene alone (related to Figure 6). Heatmap of poor survival rate with elevated 5-gene metabolic signature (TALDO1, GPI, LDHA, SHMT2, and ADK) together or each gene alone.

Supplementary Figure 11

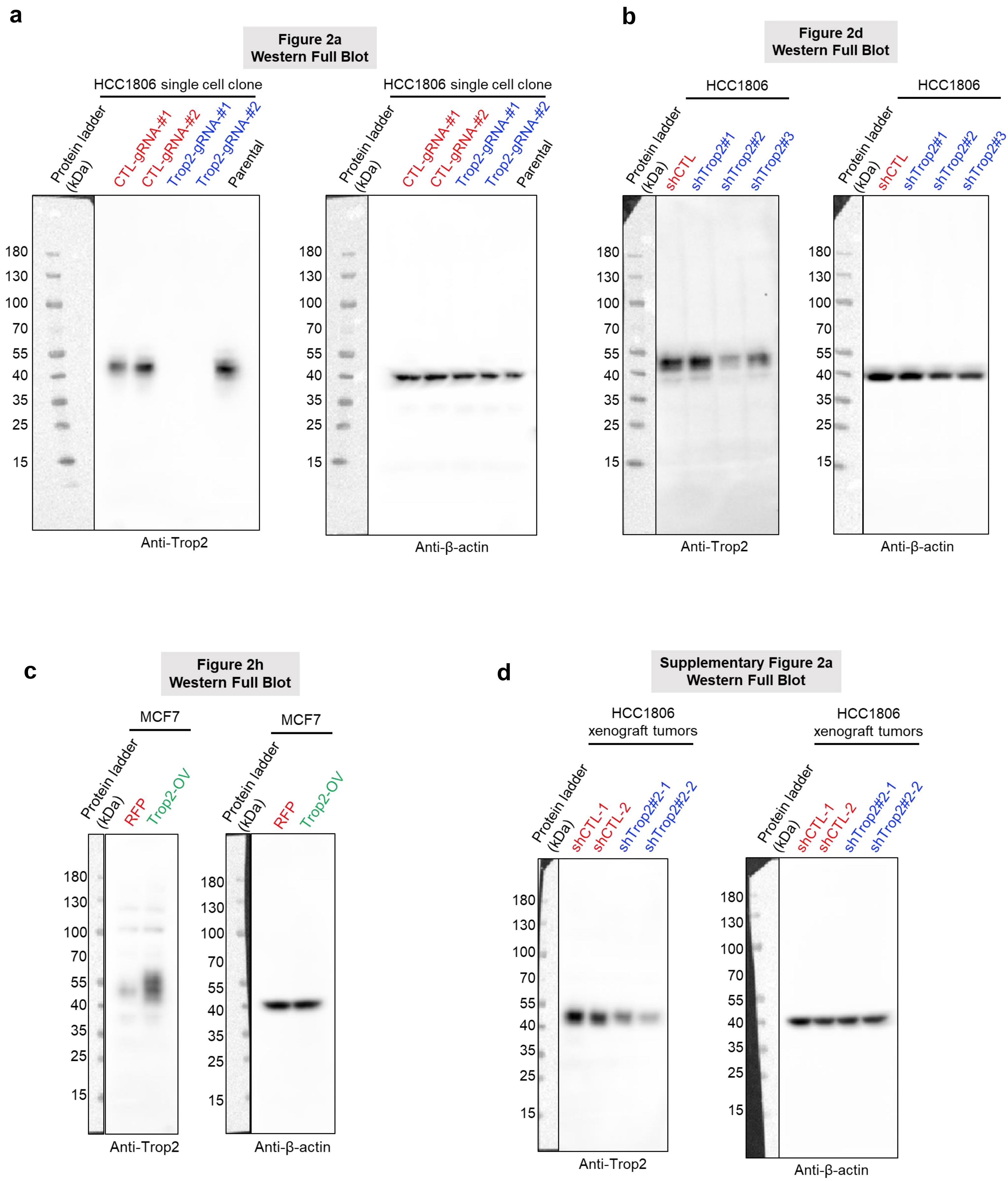

Supplementary Figure 11. Full, un-cropped images of western blots (related to Figure 2 and Supplementary Figure 2). Full, un-cropped images of western blots used in (a) Figure 2a, (b) Figure 2d, (c) Figure 2h, and (d) Supplementary Figure 2a are shown.

Supplementary Table 1. List of proteins that were upregulated or downregulated upon Trop2 knockdown in TNBC xenografts.

	HCC1806 Tumors															
Row Labels	shCTL-tumor#1 technical replicate-1	shCTL-tumor#1 technical replicate-2	shCTL-tumor#1 technical replicate-3	shCTL-tumor#2 technical replicate-1	shCTL-tumor#2 technical replicate-2	shCTL-tumor#2 technical replicate-3	shTrop2-tumor#1 technical replicate-1	shTrop2-tumor#1 technical replicate-2	shTrop2-tumor#1 technical replicate-3	shTrop2-tumor#2 technical replicate-1	shTrop2-tumor#2 technical replicate-2	shTrop2-tumor#2 technical replicate-3	Np	p-value	-log(p-value)	Average	Significance
TALDO1	2.41597729	3.53307646	3.32715352	3.4115596	2.86486436	2.54374611	-4.1276305	-3.05757	-3.5861279	-1.8400531	-1.5326923	-0.8728103	6	4.455E-08	7.35115538	-5.5188769	-40.570122
TAGLN2	1.39095262	2.83037134	2.89742828	2.60886193	3.37467381	2.98228843	-2.4460671	-2.3910654	-2.5397602	-1.6011195	-1.2012376	-0.3879965	10	3.9246E-07	6.4062046	-4.4419704	-28.456172
ACTG1	0.4487113	1.34405913	1.24566871	1.80300712	1.70330752	1.93577765	-2.1787177	-1.4853937	-1.8078387	-2.9495973	-3.4608229	-3.0992998	11	8.9482E-09	8.04826209	-3.9103669	-31.471658
LGALS3BP	1.90887474	2.85151488	3.04711986	2.86928457	3.94962877	2.62797769	-0.6929616	0.33422641	-0.8812724	0.1852596	-1.0968849	-0.0227006	13	1.5705E-05	4.80396987	-3.2381223	-15.555842
BCAP31	1.36295915	2.21653043	2.19248903	2.62961626	2.01584625	1.69866466	-3.0940683	-1.3193054	-2.3178061	0.39023836	0.12235481	-0.0431331	7	5.9239E-06	5.22739551	-3.0629709	-16.01136
NOLC1	2.57306921	1.9858549	2.30109911	3.06393837	2.83432459	1.09328808	-1.8584888	-2.0661194	-1.3004132	-0.0807355	0.61039612	0.25737006	6	4.9258E-06	5.30752676	-3.0482609	-16.178726
VASP	2.63489824	4.17600214	2.56830986	0.58691457	0.99639828	1.08489314	-0.5162191	-0.8071741	-1.0634558	-1.4465227	-0.9565859	-0.1098791	7	2.9625E-05	4.52833946	-2.8245422	-12.790486
HSP90AB1	1.47016361	1.84039669	1.67322309	1.72689535	1.60788503	1.53327535	-0.7120177	-1.3043961	-1.3391449	-1.5852524	-0.8105087	-0.5632092	8	3.0389E-07	6.51728751	-2.6943947	-17.560145
CPNE1	0.9768267	0.60084291	0.0128995	2.86095337	2.64226193	1.36071389	-1.4633596	-1.4305	-0.4840938	-1.2323325	-1.3584028	-1.3593068	9	2.7572E-05	4.55952728	-2.6304156	-11.993452
ADK	1.51648408	1.4748986	0.92632368	1.40432134	4.18378461	1.93294709	0.09932811	-1.0082134	0.40833098	-1.2193113	-1.3160015	-1.1019378	7	0.00042578	3.37081848	-2.5960941	-8.7509618
FAM129B	1.38292094	0.76926087	0.98033403	1.87935936	1.51634698	2.35018253	-1.5080963	-0.7423762	-1.9391738	-0.6786981	-0.4322927	0.27913836	7	1.4689E-05	4.83300158	-2.3166506	-11.196376
EEF1A1P5	0.37378665	0.14258342	-0.4991385	-0.0376672	1.74454415	2.0683976	0.57692982	-1.4057746	-0.3364208	-3.1021187	-3.1599883	-2.5327523	11	0.00204085	2.69018978	-2.2921052	-6.1661979
SAMHD1	0.8911154	1.16823003	0.6678703	1.47888282	1.72178613	1.45698701	-1.4253414	-0.9697274	-0.5793581	-0.8615193	-1.2253347	-1.1306562	9	1.172E-07	6.93107594	-2.2628015	-15.683649
CCT8	2.16566026	1.94001562	1.77634859	1.30012612	0.5730117	0.55392483	-1.1862636	-1.0338909	-0.7658304	-0.5640122	-1.0995703	-0.2435353	10	8.6796E-07	6.06150182	-2.200365	-13.337516
MYH4	-1.788927	-1.105392	-0.8975623	0.88883895	0.94526531	0.55070887	-5.1552226	-0.3878293	-0.4636639	-3.1709049	-3.2629698	-2.1229333	6	0.00046687	3.33080684	-2.1927426	-7.3036021
CTSB	1.13119591	1.21647278	0.70767095	1.19092048	0.54100098	1.20034405	-1.5416979	-0.881584	-1.8528587	-0.1624774	-1.0976189	-1.5410669	8	2.4483E-05	4.61113635	-2.1774848	-10.040679
HDGF	0.69855945	1.70076101	2.49910042	1.74363211	2.52895325	2.22083219	-1.0708268	-1.346667	-1.4580333	0.52963631	1.43664608	0.82390716	10	0.00013276	3.87692353	-2.0795293	-8.0621761
GPI	1.68105364	1.01726279	0.92644123	1.20952219	1.87340647	1.49853378	0.27795835	0.27771246	0.5173191	-2.0202414	-1.9669595	-1.2731686	22	1.2103E-05	4.91710237	-2.0656	-10.156766
SHMT2	1.19451711	1.38350009	0.69858966	1.4559761	2.03423779	1.1140133	-1.0189742	-1.037953	-0.7304319	-0.8189548	-0.4316882	-0.4240789	14	1.9214E-06	5.71638182	-2.0571525	-11.759469
ATP2A2	1.31623951	1.30124808	-0.0327544	0.93083472	1.11434389	0.44477189	-0.7003134	-1.2681438	-1.0551548	-1.4206587	-1.7137125	-1.034963	6	2.3707E-05	4.6251188	-2.044605	-9.4565408
CHD4	-0.3286263	0.93375729	1.62778825	1.5867822	2.81727863	2.02964314	-1.1978267	-1.514054	-0.7739213	0.14197179	0.16545936	-0.1696906	9	0.00017941	3.74616162	-2.0024475	-7.5014918
LDHA	0.79932931	0.61067971	0.85754785	2.45821909	2.58174911	1.44524619	1.76131711	1.45681423	1.50476314	-2.3695625	-3.3996811	-2.1209567	19	2.0529E-06	5.68763623	-1.9866795	-11.29951
AKR1C2	2.35985764	1.92246537	1.27580154	1.32600374	1.14202006	0.93158066	-0.140981	-0.4121091	-1.0357324	-0.3442383	-0.5302279	-0.4900118	7	1.0468E-06	5.98014669	-1.9851716	-11.871617
PRDX2	1.44064131	1.15032541	0.82571858	2.10217603	1.09542804	2.19388949	-0.623228	0.10858906	-0.8149452	-0.4938611	-0.3455722	-0.9321806	7	3.3302E-05	4.47753592	-1.9848961	-8.8874438
NPM1	1.05039677	1.2645568	1.00603709	1.22758169	1.93048251	1.78055538	-1.478938	-2.1354266	-1.7241968	0.50288014	0.23008686	1.04904982	6	4.7745E-06	5.32107438	-1.9693591	-10.479106
MYBBP1A	0.35282374	1.48660662	1.73689193	0.13170055	2.49190291	2.10226298	-0.9902924	-2.993771	-0.5727178	0.02578547	0.6472583	0.44004345	20	0.00637138	2.1957665	-1.9576471	-4.2985359
MCM5	0.88540435	0.08702714	-0.0549968	0.31205158	0.62028139	0.232303	-2.5510531	-1.7818967	-2.3900175	-0.9718933	-1.0956614	-0.8541732	7	1.4211E-06	5.84737228	-1.954461	-11.428461
BAG3	0.6120008	1.6771179	2.49600128	-0.1174796	0.81648134	1.32601765	-2.2460623	-1.8289545	-1.3451531	0.53089312	-0.2279564	0.20387999	7	0.00170509	2.7682534	-1.9539154	-5.408933
PPIB	0.86049715	0.19140873	0.38068338	1.86510516	0.90954365	-0.0535588	-0.5711285	0.27986826	-0.3857795	-2.1940915	-2.624449	-1.9470083	6	0.00056298	3.24950413	-1.9327113	-6.2803533
RSL1D1	0.02683221	0.99586895	2.50301423	1.75476527	3.17505854	1.99975801	-0.8384237	-1.7649042	-1.0081761	0.25308393	1.12055181	1.11552425	9	0.0017797	2.74965322	-1.9296069	-5.3057498
XRCC6	1.39510262	0.73707459	0.29805729	1.47241712	1.66328478	1.39722841	-0.3289685	-0.7490073	-0.8840618	-0.6951044	-1.1935615	-0.7624991	31	2.5237E-06	5.59796652	-1.9293946	-10.800686

Supplementary Table 1. List of proteins that were upregulated or downregulated upon Trop2 knockdown in TNBC xenografts.

CAT	1.61544423	1.4787201	1.21409925	1.60446634	1.32324354	1.24870694	-0.6456659	0.12998523	-0.3804708	-0.6746407	-0.754189	-0.4794081	26	2.90E-07	6.53824182	-1.8815116	-12.301778
P4HB	1.21239257	-0.0258031	-0.3057262	1.49726423	0.30960097	1.34509725	-0.648946	0.38923369	-0.9140007	-2.2097651	-2.3284256	-1.4608481	20	0.00088654	3.05230132	-1.8675962	-5.7004665
UBA6	1.8661147	1.55474341	-0.0596769	2.33362049	1.9107257	1.28628924	0.60152635	-0.8201366	-1.0362246	0.15425003	-0.5312629	-0.5860991	6	0.00060998	3.21468774	-1.8516272	-5.9524034
EZR	1.10743643	1.23844147	0.86655726	1.6879029	1.1040754	1.01301072	-1.7666729	-0.5500953	-1.1860796	-0.2476805	0.01966301	-0.2640191	11	1.2591E-05	4.89994859	-1.8353848	-8.993291
ANP32A	1.37308056	1.0091989	1.06337787	1.58683006	1.7461701	0.56388012	-0.7343602	-0.166476	-0.8987695	-1.027002	0.06025762	-0.6860764	8	4.2407E-05	4.37255772	-1.7991607	-7.8669339
NEB	0.26193545	1.18683427	0.11576556	0.44620754	0.96560631	1.23491807	-1.6577296	-2.3299492	-1.7504551	-0.0275832	-0.4652649	-0.1521615	6	5.1335E-05	4.28958543	-1.7657351	-7.5742717
MTDH	0.14489072	0.39829913	0.36937823	0.48278236	1.20223036	1.31167674	-1.0955965	-0.369791	-0.6594174	-2.0443783	-0.9053129	-1.0290837	8	6.9857E-05	4.15578826	-1.6688062	-6.9352053
NAT10	0.88219433	0.99265608	-0.1069159	0.5822346	0.3129521	0.9038733	-0.9595883	-1.8772127	-0.3722405	-1.1142185	-1.4379078	-0.4292816	6	0.00040205	3.39572512	-1.6262407	-5.5222663
HSPH1	1.15754999	1.66333108	1.03677499	1.3567439	0.79176031	0.91438618	-0.3410886	0.16175599	0.02671338	-1.0590388	-0.7180971	-0.8220108	10	3.7157E-06	5.42996108	-1.6120521	-8.75338
NCL	0.7389494	1.26538351	1.18617612	0.96137303	1.02573009	1.01979896	-1.5286157	-1.00494	-0.7977414	-0.4600487	0.26299074	0.14067165	23	1.3893E-05	4.8571917	-1.5975158	-7.7594402
FAM213A	1.11377887	0.66247246	0.478695	0.8356151	-0.3017522	0.93014986	-1.4431693	-0.4154712	-1.015548	-0.7566193	-1.1997955	-0.8512251	6	0.0002269	3.64416621	-1.5667979	-5.7096721
HNRNPM	0.63125984	1.82406937	0.42632585	2.04221027	-0.3223898	0.06493646	-3.0897882	-2.1228403	-2.7567177	1.4290493	1.34179923	0.65472983	9	0.00116192	2.93482381	-1.53503	-4.5050425
EEF1G	0.64773533	1.33276863	1.32494198	0.66210985	1.97100123	0.27587834	-0.5274189	-0.3644926	-0.9563453	-1.2471102	0.15523112	0.14192799	6	0.00053983	3.2677447	-1.5021072	-4.9085028
QARS	1.10940038	0.75899353	1.06477994	0.03932231	0.61834402	1.19206407	-0.8696858	-1.6181043	-1.0564603	0.13209313	-0.295211	-0.3002969	10	9.9884E-05	4.00050234	-1.4650949	-5.8611156
AKR1C1	0.92930458	2.08377806	0.48927806	1.85801262	0.5028636	0.73618554	0.09602787	0.54071541	0.19239237	-1.0697382	-0.9161292	-0.6540212	8	0.00171642	2.76537728	-1.4016959	-3.8762179
ANXA11	0.10849584	0.50953241	0.69354538	1.11098622	1.34929665	1.13994636	-0.0271671	-1.4421375	-0.6192058	-0.3071034	-0.4293239	-0.3611081	6	2.9533E-06	5.52968564	-1.3496414	-7.4630929
CCT3	1.21091424	0.75700374	1.11212018	0.9629854	1.02288969	0.87952521	0.57151024	0.05545714	0.69756014	-1.03741	-0.9858757	-1.4448621	6	1.0827E-05	4.96549895	-1.3481765	-6.6943687
NUMA1	0.83211116	1.06356208	0.74602596	0.67202966	0.97082775	0.53236255	-0.674182	-2.1704365	-0.2990698	-0.0319719	-0.1917394	0.11563568	22	0.00024271	3.61491803	-1.3447805	-4.8612714
TLN1	0.81763834	1.19199059	-0.5708442	2.01638362	2.04166627	1.40186792	-0.9188143	-1.2087306	-1.2447804	1.00424827	0.33677552	0.94584539	7	0.00102174	2.99066004	-1.3306931	-3.9796508
LAMC2	1.06407777	0.72981386	0.87480011	0.95419633	0.94081323	0.23000419	-1.735947	-2.2741461	-0.922689	0.69681224	0.51139752	0.57447849	7	0.00220821	2.65595866	-1.3239666	-3.5164004
HIST2H3A	0.51929959	0.82493081	0.49806842	1.05370714	0.60524716	0.11066122	-1.1660266	-0.3188365	0.21304851	-1.008467	-0.9389014	-0.8669161	6	0.00034466	3.46261095	-1.2830022	-4.4425376
HADHA	0.5160337	-0.0776415	-0.300626	0.99496251	1.03027206	1.03714104	-0.4634308	0.295171	-0.169304	-1.5344994	-1.2858135	-1.0244748	26	6.5001E-05	4.18707952	-1.2304156	-5.1518478
CCT2	0.47585414	0.43725875	-0.439832	0.71136703	0.84003825	0.06294876	-0.5506591	-0.1227021	-0.231926	-1.2463444	-1.5501567	-1.5002797	7	0.00036548	3.43713318	-1.2149505	-4.1759466
HSPA1A	0.72498552	0.53075065	-0.1128531	0.87050185	1.30422152	1.72796667	0.6600449	0.65928007	0.43410936	-1.6521959	-1.3172044	-0.8124436	28	0.00087472	3.0581318	-1.1789971	-3.6055286
PDIA4	0.37760292	0.71503991	0.4286468	1.32070263	1.12507893	0.16597162	-1.9312031	-2.46845	-1.1540064	0.51199833	1.34036283	0.82058311	20	0.00383927	2.41575091	-1.1689597	-2.8239154
DDX21	1.1147468	1.17946789	0.89328418	0.19336452	0.85308921	0.48996807	-1.2132742	-1.808482	-1.1301791	0.55030116	0.60627839	0.86992068	16	0.00021822	3.66110649	-1.1415593	-4.1793701
FASN	0.6304411	1.01503842	0.70035089	-0.0559759	0.63147201	0.65829844	-0.1476923	-0.7956668	-0.6169078	-0.0902933	-0.9795582	-0.5454562	54	0.00023696	3.62532184	-1.1258666	-4.0816287
CCT6A	0.82890782	0.18281285	-0.2495964	0.52648834	1.23568901	1.39265022	0.91652775	1.43162797	0.2063276	-2.1003327	-1.7403078	-1.3837442	9	0.00032018	3.49460695	-1.0978089	-3.8364105
PKM	0.07832307	-0.0579022	0.66408387	1.29247688	2.40996462	1.67223116	0.85372706	0.56732178	-0.2894053	-0.9594187	-0.7135283	0.06677207	17	0.00491263	2.3086863	-1.0889515	-2.5140473
GOT2	1.10393864	0.85302242	0.6258546	0.53134811	0.48399114	0.30435721	-1.1852688	-0.3660641	-0.8755445	0.34557055	-0.2195264	-0.3012318	7	0.00030493	3.51579594	-1.0840962	-3.811461
HP1BP3	0.83889746	1.18599771	1.11771175	0.76456485	0.80401537	0.83837099	-0.318327	-0.2915765	-0.3179846	-0.4359397	0.23696014	0.32532343	6	7.5566E-06	5.12167277	-1.0585171	-5.421378
EPPK1	0.45388294	0.43896296	0.1463327	0.77798043	1.30924769	1.29150919	0.42941713	-0.8881404	-0.4143202	-0.4934779	-0.2535515	-0.0169896	23	0.0006274	3.20245407	-1.0091631	-3.2317983
ALDH3A2	1.40944255	1.34415781	0.32094329	0.49187238	1.06899847	0.84256537	-0.3953595	0.3377469	0.56393893	-0.5331985	-0.6148208	0.0673446	9	0.00175102	2.75670818	-1.0087214	-2.7807505
VIM	-0.9507555	-1.0034062	-1.1105638	-0.4539277	-1.6218804	-1.3258462	0.31220412	0.78000032	0.35696265	-0.2658377	-0.6273	-0.8511615	10	0.00014927	3.82603563	1.0285413	3.93523566
CROCC	-0.9424984	-1.4315583	-1.0955088	-0.2260783	-0.9029161	-0.4458296	0.3519489	-0.1034418	-0.0994212	0.38090296	0.0258176	0.67042142	7	0.00014199	3.84773196	1.0451029	4.02127582
MDH1	-0.326145	-1.2880537	0.03982029	0.09295907	-0.6754106	-0.0485851	1.76762613	1.89042632	1.47426995	-0.08845	0.16157177	-1.1141286	6	0.00446696	2.34998756	1.04945509	2.46620642

Supplementary Table 1. List of proteins that were upregulated or downregulated upon Trop2 knockdown in TNBC xenografts.

ALDH1A1	-0.1475397	-0.8508591	-0.0941785	-1.288327	-1.7627418	-1.2656691	1.27143551	1.23385515	0.4844687	-0.8867796	-0.1189518	-0.832969	15	0.00090269	3.04446115	1.09339572	3.32880078
NUP153	-0.4166496	-0.4629211	0.42807558	-0.6092747	-0.2257773	-0.8621629	0.2002325	-0.7116813	-0.4535586	1.31758002	2.00141707	2.11147748	7	0.00106816	2.97136365	1.10236286	3.27552094
PRRC2A	-0.0219635	-0.7694169	-1.0619523	-0.0223772	-0.7078586	-0.948604	-1.8548096	-1.9883955	-1.3136346	2.78680365	3.53439911	1.92943623	6	0.00014961	3.82504355	1.10432863	4.22410508
TPD52L2	-1.0148739	-0.5991733	-0.4189812	-1.8218996	-0.7458641	-0.7012001	0.38741635	0.17757574	1.07088891	0.04285713	-0.1416625	0.08417491	7	0.0008388	3.0763429	1.1538738	3.54971147
PRDX3	-2.0110888	-1.8101638	-1.0913489	-1.2753016	-1.545548	-1.2755139	1.87086462	0.89569901	0.7304946	-1.6994217	-2.2695764	-1.4545168	10	0.00055529	3.25547903	1.18041804	3.84282619
MARS	-0.5712896	-1.0405097	-0.3279386	-1.3769921	-1.2182089	-0.954625	0.31182012	0.47964483	0.72985003	-0.157635	0.4399223	-0.0350832	13	3.9963E-05	4.3983405	1.20968048	5.32058667
GSN	-0.6281956	-1.0864525	-0.1229948	-0.7681454	-0.309848	-0.4507451	2.5211783	1.06436238	0.31304544	-0.1290367	0.27099389	-0.0335146	7	0.00212024	2.67361599	1.22890171	3.28561125
CCT5	-0.7225692	-0.6801768	-1.3280442	-0.9564386	-2.0586402	-1.6077504	0.87455603	1.1370931	1.13570722	-0.7672581	-0.8492805	-1.1205623	14	0.0001339	3.87320693	1.29397915	5.01184901
MYH2	-1.874665	-1.4013806	-1.0031299	-1.5239016	-0.7214462	-1.4230962	0.63634563	0.40358614	-0.0905751	0.10309544	-0.2195542	-0.9959664	6	0.00066935	3.17434603	1.29742517	4.11847645
AHNAK	-0.5063758	-1.0231869	-0.6749779	-0.6351022	-1.3974336	-0.9269893	-0.3973786	0.3658113	-0.0441168	1.04936441	1.14846534	0.66203594	223	0.00010893	3.96283463	1.32470787	5.24959821
IQGAP1	-1.2811937	-0.9822384	-0.5897586	-0.7608518	-1.8018272	-1.0195979	2.05802599	1.37662082	0.61089648	-0.4426509	-1.4008908	-0.4091775	29	0.00293732	2.53204872	1.37138193	3.47240587
GDI2	-1.3496343	-1.8179858	-1.5592936	-0.9239483	-1.340102	-1.3057342	0.66737947	1.53086358	0.13683839	-0.2831916	-1.0420741	-1.0603576	11	0.00129899	2.88639545	1.37435938	3.96694467
DHX9	-1.4012703	-1.4268729	-1.543709	-0.6411211	-0.803102	-0.2575649	0.26362248	0.1671928	0.42373125	0.28335061	0.99325932	0.04865915	21	1.3279E-06	5.87683114	1.37557594	8.08402753
HSPD1	-1.2030644	-0.9849702	-1.0713278	-1.5968733	-1.1268876	-1.1587676	2.18405199	1.56149247	1.17592869	-1.1262068	-2.1519536	-0.3409762	20	0.00071895	3.14330206	1.40737123	4.42379288
RRBP1	-0.4934708	-0.9752642	0.12036349	-0.970778	-1.03586	-0.8687651	-0.5124497	-0.4541434	0.51517919	1.03433185	2.55858327	1.34426908	22	0.00280771	2.55164807	1.45159082	3.70394892
FLNB	-0.9053477	-0.9943574	-0.1142228	-0.1872129	-1.8356809	-0.7632269	0.58319636	0.56450674	0.18307244	1.09588609	1.2157203	0.45209133	27	0.00059276	3.22711929	1.48242032	4.78394719
ANXA2	-0.4597762	-0.508089	-0.8752775	-0.0807438	-1.3287801	-1.1509133	0.41259501	1.18189987	-0.4454228	1.10273772	1.53608183	0.94391827	13	0.00096649	3.01480357	1.52256497	4.5902343
RCC1	0.00715409	-0.8487536	-0.3969391	-1.345094	-1.4967879	-1.3601743	1.70497756	1.70169597	1.31180076	-0.4468556	-0.4422969	-0.0116365	6	4.0256E-06	5.39517226	1.54304669	8.32500271
PAICS	-1.2002744	-2.1796943	-1.473926	-1.0673112	-2.4895351	-1.8282193	1.37014733	0.99521473	0.9110683	-1.1848853	-1.422248	-1.3224736	7	0.0003455	3.46155193	1.59763062	5.53028137
SORD	-1.844065	-1.5708957	-1.4628461	0.09038022	-1.6377584	-1.503455	1.69926393	1.56408482	2.46612758	-1.6155934	-0.6382381	-1.5712846	7	0.00021787	3.66179582	1.63883337	6.00107317
G3BP1	-0.6650733	-1.0808173	-1.272667	-0.5052922	-2.0228566	-0.6661732	-0.2071249	0.48864365	0.53145315	1.36549369	1.0524644	0.48228386	6	0.00026547	3.57597809	1.6543489	5.91591543
GAPDH	-0.9794866	-2.0620965	-1.5726908	-1.7295762	-1.5292283	-0.7677949	2.34200439	2.15091034	0.97338528	-1.5805366	-1.8218958	-0.7173269	23	0.0030077	2.52176575	1.664569	4.19765309
TMPO	-0.3201313	-1.6880103	-1.1888718	-0.3724946	-0.8801404	-0.725975	0.86369597	0.1410226	1.08715553	0.33768561	1.39039575	1.21282619	11	0.00023475	3.62939118	1.70140085	6.17504922
DSG2	-2.3030487	-2.5046026	-0.7712189	-2.2216537	-2.8487306	-0.7957393	1.44025597	0.24960169	0.53065642	-0.8479677	-1.6328707	-0.8554106	17	0.0044789	2.34882862	1.72154317	4.04360987
ACTN4	-0.3142345	-1.5499427	-0.6660334	-1.3469131	-2.0429116	-0.9763084	0.19939959	1.20921511	0.07230395	0.83633779	0.63677907	0.4856901	10	0.00018237	3.73904352	1.72267821	6.44116881
ANXA1	-0.8081599	-1.805513	-1.4097116	-0.7515575	-2.0674545	-1.4813612	0.27168537	0.62322559	0.1050281	0.55704394	0.3385346	0.18894216	25	7.28E-05	4.13786976	1.73470291	7.17797473
BCLAF1	-1.0440102	-0.8159437	-0.523881	-0.790351	-0.9090324	-0.5606392	0.83034434	1.51913847	0.24926774	0.79229492	1.04083015	1.48209568	7	1.6543E-05	4.78138119	1.75963814	8.41350071
LARS	-1.4043413	-1.049889	-0.7619898	-1.4159035	-0.9043648	-1.1410854	1.48863015	1.44188734	1.13739217	0.17680801	-0.1109292	-0.0264475	7	8.5369E-07	6.0686997	1.79748579	10.9084015
ACBD3	-1.2398802	-1.6918393	-1.6669481	0.39851416	-1.428374	-1.6514115	-0.2209118	-0.2634142	1.57489151	1.46895977	0.48453189	0.71871645	6	0.00255972	2.59180819	1.84045208	4.77009878
TNC	-1.7493248	-2.4454955	-2.8219892	-2.254466	-2.490311	-1.851524	0.76452535	0.94380003	0.55658859	-1.0969378	-2.1139981	-1.2861211	48	9.5191E-05	4.02140409	1.89682788	7.6279114
PTBP1	-1.6382822	-0.7229893	-1.232873	-0.9712928	-2.4224557	-2.9218632	1.71147161	1.87671837	1.60066307	-1.0854228	-1.1129171	-1.5155742	13	0.00010051	3.99778397	1.89744922	7.58559207
CLIC1	-1.5874826	-0.5608649	-2.1538849	-2.3378371	-0.5955738	-0.7411721	2.25815459	1.2394261	1.10295452	-0.1715846	-0.4199723	-0.5167614	8	0.00152829	2.81579362	1.9115054	5.38240471
HADHB	-1.005437	-1.2325155	-1.2440735	-1.1082437	-1.6399129	-0.8960276	0.99731357	1.58073903	1.40200173	0.15349537	0.27215308	-0.0429139	12	9.6435E-07	6.01576399	1.91483318	11.5191845
EEF1B2	-1.7210454	-2.1153667	-1.2063933	-1.9118965	-1.1172341	-1.4155481	1.60762055	0.45358326	1.02505845	-0.4727984	-0.2286829	-0.2895696	6	2.8034E-05	4.55231415	1.93044924	8.78801141
COL18A1	-0.7703436	-1.6875745	-0.4980778	-1.5812183	-1.7179512	-1.6210419	1.19862883	0.79876245	0.94162235	0.29753989	0.38090825	0.16414343	7	9.0609E-08	7.04283001	1.94296874	13.6839986
GOT1	-2.0686667	-1.4038679	-1.1901405	-2.6336861	-0.831726	-1.2568875	0.34446808	2.48087006	0.5000767	-0.4201093	-0.1053109	-0.345204	9	0.00181247	2.74172987	1.9732942	5.41023964

Supplementary Table 1. List of proteins that were upregulated or downregulated upon Trop2 knockdown in TNBC xenografts.

FLNA	-1.9791659	-2.4911905	-1.7291281	-1.5592676	-1.95403	-1.3841803	-0.044846	0.02837591	-0.0333982	0.71277471	0.21660799	-0.1034598	28	2.2756E-06	5.6429092	1.97883615	11.1663927
AIFM1	-1.9816907	-0.9614079	0.85459458	-2.0381602	-1.905117	-0.9963347	1.37846806	2.00443035	1.57800876	-0.373262	0.59124419	0.33082478	7	0.0006952	3.15788712	2.08963835	6.59884203
PFN1	-1.2573299	-2.0169485	-1.5715483	-2.0887463	-2.7740702	-1.5494565	1.21428449	0.44528784	0.62122178	-0.1811831	-0.3269194	-0.3895967	9	1.2245E-05	4.91203879	2.10686576	10.3490064
BASP1	-2.8528619	-2.1527465	-0.6016897	-0.6297752	0.00743098	-0.1832325	0.65252865	-0.213618	-0.8937275	2.56202603	2.57483363	1.72647275	10	0.00016239	3.78944377	2.13689838	8.09765625
C1QBP	-2.0216577	-2.5268939	-1.8474369	-2.1027043	-2.5874438	-2.9199923	1.75870237	0.18627429	0.64596232	-1.3536931	-1.4517409	-0.8535419	8	4.9778E-05	4.30296321	2.15634865	9.2786889
SMARCA4	-2.1583276	-1.2433531	-1.2919246	-2.1860842	-1.0939311	-0.5365	0.14296328	0.75202293	1.07039676	0.75484265	1.17117368	0.54710002	6	8.2196E-05	4.08514789	2.15810332	8.81617124
TPI1	-1.3051308	-1.7819592	-1.2151895	-0.9965425	-1.5527163	-1.6514699	1.6236326	1.134824	1.48182823	0.24303581	0.14913211	-0.1481841	6	7.7173E-07	6.11253631	2.16454615	13.230867
DSC3	-1.6690317	-0.3338609	0.46660097	-2.8004338	-0.7847032	-0.7804923	0.32760641	0.91291956	0.62023947	2.17429605	2.43909779	0.69435197	6	0.00312872	2.50463307	2.17840538	5.45610616
CAD	-1.3346079	-0.6768954	-1.935009	-0.0900658	-0.9560605	-1.1616625	1.16919198	1.43302362	0.37328892	1.93178734	0.76731587	1.35497943	10	9.964E-05	4.00156476	2.19731471	8.79269709
TRIM29	-0.0240117	-0.8132817	-1.3344435	0.5219091	-0.8355956	-1.4488048	1.50933159	2.44797316	1.45491007	1.37530696	1.3143281	1.16850822	12	0.00023524	3.62849139	2.20076437	7.98545456
ATP5O	-0.4581525	-1.0312886	-1.5145799	-2.9608339	-2.4540503	-1.5186417	1.53513483	2.85823301	1.8093131	-0.7348009	-0.8283583	-1.1411145	6	0.00012202	3.91355264	2.23932568	8.76371894
IARS2	-0.8646389	-1.0346046	-1.3427601	-1.5010594	-2.3302676	-1.7962959	0.33977642	1.06176572	0.76867895	1.99162999	1.60104719	1.18830469	7	9.97E-07	6.00130498	2.63680491	15.8242704
RTN4	-1.3313878	-1.5759522	-2.0965939	-2.9850719	-3.3010079	-0.9617905	1.60465361	-0.1494841	0.53440507	1.20095738	0.56667192	0.46310106	6	0.00020508	3.68808069	2.74535151	10.1250779
CALR	-2.4319574	-2.839013	-2.4245375	-0.7686305	-2.1248705	-2.1739369	1.66117674	0.92441359	1.59312968	0.30291526	-0.2250231	-0.4838351	17	5.1088E-06	5.29168452	2.75595379	14.583638
FUBP3	-1.8721046	-2.1704808	-1.1836352	-2.0954343	-1.3938954	-1.9730031	0.50066665	0.43776378	1.65729163	1.66702062	0.69795409	1.22761748	8	6.3426E-06	5.19773457	2.81281127	14.6202464
SLC25A3	-1.7945734	-2.1367874	-0.9721683	-1.2361918	-2.8309661	-3.5673868	1.54978082	0.63829954	0.87698053	0.65732901	0.71616786	0.33503246	6	4.2456E-05	4.37206595	2.88527735	12.6146229
CAST	-1.8730503	-1.6742304	-1.1828174	-2.3077395	-1.4434522	-1.1374921	0.79845273	2.01457064	1.08850122	1.62764691	2.25859678	0.20314883	30	3.0951E-05	4.50932318	2.93494983	13.2346373
FABP5	-1.7861166	-1.6286718	-0.8067726	-1.4729329	0.00486169	-1.098987	2.37999588	3.42914057	1.99104244	1.15250279	1.58912256	0.30317939	14	5.1861E-05	4.28516275	2.9389338	12.5938097
GSTP1	-2.1399842	-2.2949315	-1.5835765	-2.9536209	-3.7161151	-2.2797891	1.50276805	1.00633368	1.15049715	0.29634429	0.30622581	0.14579766	27	3.7397E-07	6.42715872	3.22933066	20.7554207
CDH1	-1.9873081	-2.8039221	-2.1812196	-3.6264247	-2.7600708	-2.6208893	2.48544719	2.60114117	2.13194459	-0.4028647	-0.5449361	-0.9515342	7	1.9895E-07	6.7012669	3.54983877	23.788417
Trop2	1.05583537	0.49831918	0.53005653	1.24482341	0.76655029	0.81078087	0.02516957	0.72899298	-1.0091305	-0.1824754	-0.231808	-0.1513922	3	0.00040787	3.38947891	-0.9545015	-3.2352628
