# Supplementary material for: Oncogene-mediated metabolic gene signature predicts breast cancer outcome
Source: NPJ Breast Cancer. 2021 Oct 28;7:141. doi: 10.1038/s41523-021-00341-6 (PMC8553750; doi:10.1038/s41523-021-00341-6)
Supplement: Supplementary file 2 — Reporting Summary [file 41523_2021_341_MOESM2_ESM.pdf]

nature portfolio

Corresponding author(s): Tanya Stoyanova

Last updated by author(s): 09/15/2021

Reporting Summary

Nature Portfolio wishes to improve the reproducibility of the work that we publish. This form provides structure for consistency and transparency in reporting. For further information on Nature Portfolio policies, see our Editorial Policies and the Editorial Policy Checklist.

Statistics

For all statistical analyses, confirm that the following items are present in the figure legend, table legend, main text, or Methods section.

n/a	Confirmed	
☐	☒	The exact sample size (n) for each experimental group/condition, given as a discrete number and unit of measurement
☐	☒	A statement on whether measurements were taken from distinct samples or whether the same sample was measured repeatedly
☐	☒	The statistical test(s) used AND whether they are one- or two-sided<br>*Only common tests should be described solely by name; describe more complex techniques in the Methods section.*
☐	☒	A description of all covariates tested
☐	☒	A description of any assumptions or corrections, such as tests of normality and adjustment for multiple comparisons
☐	☒	A full description of the statistical parameters including central tendency (e.g. means) or other basic estimates (e.g. regression coefficient) AND variation (e.g. standard deviation) or associated estimates of uncertainty (e.g. confidence intervals)
☐	☒	For null hypothesis testing, the test statistic (e.g. F, t, r) with confidence intervals, effect sizes, degrees of freedom and P value noted<br>*Give P values as exact values whenever suitable.*
☒	☐	For Bayesian analysis, information on the choice of priors and Markov chain Monte Carlo settings
☐	☒	For hierarchical and complex designs, identification of the appropriate level for tests and full reporting of outcomes
☒	☐	Estimates of effect sizes (e.g. Cohen's d, Pearson's r), indicating how they were calculated

Our web collection on statistics for biologists contains articles on many of the points above.

Software and code

Policy information about availability of computer code

Data collection

Acquisition of gene expression and clinical data:
The mRNA z score data, overall survival information, ER/HER2/PR status, and tumor stages of METABRIC and TCGA Firehose Legacy clinical datasets, which were imported from the original TCGA Data Coordinating Center via the Broad Firehose (https://gdac.broadinstitute.org/, doi:10.7908/C11G0KM9) were obtained from cBioPortal for Cancer Genomics (https://www.cbioportal.org/). The mRNA raw data and ER/HER2/PR status in E-MTAB-365, and GSE21653 clinical datasets were used to generate the Kaplan-Meier Plotter (https://kmplot.com/analysis/index.php?p=service) and then normalized to z score based on equation: (raw score- population mean)/population standard deviation. Heatmaps of mRNA z-score in Figure 3 were generated via Morpheus (https://software.broadinstitute.org/morpheus/). Relapse-free survival information of Kaplan-Meier plots of E-MTAB-365 (n=426), GSE21653(n=230), GSE2034 (n=286), GSE20685 (n=327), GSE20711(n=88), GSE17705 (n=196), GSE45255 (n=94), GSE1456 (n=159), GSE3494 (n=249), GSE12276 (n=204) were collected from Kaplan-Meier Plotter (https://kmplot.com/analysis/index.php?p=service) and then Log Rank Test of survival analysis was performed in Prism software. N numbers indicate the number of patients with the follow up survival data available of the datasets.

Patient cohorts: METABRIC includes 1355 ER+ and 299 TNBC samples, TCGA contains 806 ER+, and 115 TNBC samples, E-MTAB-365 includes 312 ER+ and 52 TNBC samples and GSE21653 includes 132 ER+ and 85 TNBC samples. The mRNA z-scores for the indicated genes from the clinical datasets were downloaded and heatmaps were generated as a comparison of ER+ breast cancer and TNBC.

Data analysis

The differences between population proportions were calculated by z-score test for two population proportions. In vitro and in vivo functional assays unless otherwise mentioned were analyzed by two tailed Student's t test. Adjusted P value of gene expression levels between ER+ subtype versus TNBC subtype was calculated based on Bonferroni testing in Prism software. Survival analysis was calculated based on Log-Rank (Mantel-Cox) Test in Prism software.

For manuscripts utilizing custom algorithms or software that are central to the research but not yet described in published literature, software must be made available to editors and reviewers. We strongly encourage code deposition in a community repository (e.g. GitHub). See the Nature Portfolio guidelines for submitting code & software for further information.

Data

Policy information about availability of data

All manuscripts must include a data availability statement. This statement should provide the following information, where applicable:

- Accession codes, unique identifiers, or web links for publicly available datasets
- A description of any restrictions on data availability
- For clinical datasets or third party data, please ensure that the statement adheres to our policy

The datasets generated during and/or analyzed during the current study are either included in the manuscript or available from the corresponding author on reasonable request.

Field-specific reporting

Please select the one below that is the best fit for your research. If you are not sure, read the appropriate sections before making your selection.

☒ Life sciences ☐ Behavioural & social sciences ☐ Ecological, evolutionary & environmental sciences

For a reference copy of the document with all sections, see nature.com/documents/nr-reporting-summary-flat.pdf

Life sciences study design

All studies must disclose on these points even when the disclosure is negative.

Sample size	Patient cohorts: METABRIC includes 1355 ER+ and 299 TNBC samples, TCGA contains 806 ER+, and 115 TNBC samples, E-MTAB-365 includes 312 ER+ and 52 TNBC samples and GSE21653 includes 132 ER+ and 85 TNBC samples. The mRNA z-scores for the indicated genes from the clinical datasets were downloaded and heatmaps were generated as a comparison of ER+ breast cancer and TNBC.
Data exclusions	None
Replication	All in vitro experiments were performed three times. Each experiment had technical triplicates. For the in vivo studies, we used n=10 for each tumor group. For liquid chromatography and mass spectrometry analysis, we used two different tumors per group and each tumor was run in technical triplicates.
Randomization	Not applicabe
Blinding	The tissue microarray (TMA) in Figure 1 was purchased from Biomax (BR1505c) including 150 breast invasive ductal carcinoma cores from 75 different patients with duplicate cores per patient. The TMA had information regarding the clinical stages and IHC results of ER, PR, and HER2 hormone receptors. Patient samples were categorized based on their hormone receptor expression levels. Each case had two different cores that were blind scored and averaged for Trop2 staining intensity. 10 out of 75 patient samples had different hormone receptor levels in each patient core. TMA has total n=22 ER+ HER2-, n=35 HER2+ (27 HER2+ ER-, and 8 HER2+ ER+), and n=28 TNBC cases. The intensity of Trop2 staining was blind scored from 0 up to 3 as shown in Figure 1A.

Reporting for specific materials, systems and methods

We require information from authors about some types of materials, experimental systems and methods used in many studies. Here, indicate whether each material, system or method listed is relevant to your study. If you are not sure if a list item applies to your research, read the appropriate section before selecting a response.

Materials & experimental systems

n/a	Involved in the study	
☐	☒	Antibodies
☐	☒	Eukaryotic cell lines
☒	☐	Palaeontology and archaeology
☐	☒	Animals and other organisms
☒	☐	Human research participants
☐	☒	Clinical data
☒	☐	Dual use research of concern

Methods

n/a	Involved in the study	
☒	☐	ChIP-seq
☒	☐	Flow cytometry
☒	☐	MRI-based neuroimaging

Antibodies

Antibodies used	For immunoblotting, we used Anti-Trop2 biotinylated antibody BAF650 (R&D), anti-transaldolase antibody (H-4) sc-166230, anti-LDH-A antibody (E-9) sc-137243, anti-mSHMT antibody (F-11) sc-390641, anti-ADK antibody (H-1) sc-514588, and anti-GPI Antibody (H-10) sc-365066 were used at 1:1000 dilution.

	For IHC, we used Anti-Trop2 B9 sc-376746 (Santa Cruz) antibody was used in 1:100 dilution for TMAs IHC and anti-human Trop2 biotinylated antibody BAF650 (R&D) in 1:50 dilution was used for xenograft IHC staining. Antibodies against transaldolase (H-4) sc-166230, LDH-A (E-9) sc-137243, mSHMT (F-11) sc-390641, and ADK (H-1) sc-514588 from Santa Cruz Biotechnology, and GPI (#A6916) from ABclonal were used in 1:100 dilution.
Validation	The antibodies were used according to the manufacturers' recommendation.

Eukaryotic cell lines

Policy information about cell lines

Cell line source(s)	MCF7 was a kind gift from Dr. James Brooks's laboratory at Stanford University (Palo Alto, CA) and HCC1608 cells were purchased from ATCC.
Authentication	All cells were authenticated through the Genetica cell line testing.
Mycoplasma contamination	Cells were tested for mycoplasma using Lonza Mycoalert Detection Kit (Lonza). All cells used in the study were mycoplasma negative.
Commonly misidentified lines (See ICLAC register)	None

Animals and other organisms

Policy information about studies involving animals; ARRIVE guidelines recommended for reporting animal research

Laboratory animals	For all the studies, 6-8-week-old female NSG (NOD-SCID-IL2Rγ-null) mice (Jackson Laboratory) were used and housed at Stanford University animal facilities.
Wild animals	None
Field-collected samples	None
Ethics oversight	All the procedures performed in this study were approved by Stanford Administrative Panel on Laboratory Animal Care (APLAC), IACUC.

Note that full information on the approval of the study protocol must also be provided in the manuscript.

Clinical data

Policy information about clinical studies

All manuscripts should comply with the ICMJE guidelines for publication of clinical research and a completed CONSORT checklist must be included with all submissions.

Clinical trial registration	Not applicable
Study protocol	Not applicable
Data collection	Not applicable
Outcomes	Not applicable
